# Global mean potassium intake: a systematic review and Bayesian meta-analysis

**DOI:** 10.1007/s00394-023-03128-6

**Published:** 2023-03-08

**Authors:** Catriona Reddin, John Ferguson, Robert Murphy, Aoibhin Clarke, Conor Judge, Vincent Griffith, Alberto Alvarez, Andrew Smyth, Andrew Mente, Salim Yusuf, Martin J. O’Donnell

**Affiliations:** 1 HRB-Clinical Research Facility, National University of Ireland Galway, Galway University Hospital, Newcastle Road, Galway, H91YR71 Ireland; 2 Wellcome Trust–HRB, Irish Clinical Academic Training, Galway, Ireland; 3 Population Health Research Institute, Hamilton Health Sciences and McMaster University, Hamilton, Ontario Canada

**Keywords:** Potassium, Meta-analysis, Nutrition

## Abstract

**Purpose:**

Increasing potassium intake, especially in populations with low potassium intake and high sodium intake, has emerged as an important population-level intervention to reduce cardiovascular events. Current guideline recommendations, such as those made by the World Health Organisation, recommend a potassium intake of  > 3.5 g/day. We sought to determine summary estimates for mean potassium intake and sodium/potassium (Na/K) ratio in different regions of the world.

**Methods:**

We performed a systematic review and meta-analysis. We identified 104 studies, that included 98 nationally representative surveys and 6 multi-national studies. To account for missingness and incomparability of data, a Bayesian hierarchical imputation model was applied to estimating summary estimates of mean dietary potassium intake (primary outcome) and sodium/potassium ratio.

**Results:**

Overall, 104 studies from 52 countries were included (*n* = 1,640,664). Mean global potassium intake was 2.25 g/day (57 mmol/day) (95% credible interval (CI) 2.05–2.44 g/day), with highest intakes in Eastern and Western Europe (mean intake 3.53g/day, 95% CI 3.05–4.01 g/day and 3.29 g/day, 95% CI 3.13–3.47 g/day, respectively) and lowest intakes in East Asia (mean intake 1.89 g/day; 95% CI 1.55–2.25 g/day). Approximately 31% (95% CI, 30–41%) of global population included have an estimated potassium intake  > 2.5 g/day, with 14% (95% CI 11–17%) above 3.5 g/day.

**Conclusion:**

Global mean potassium intake (2.25 g/day) falls below current guideline recommended intake level of  > 3.5 g/day, with only 14% (95% CI 11–17%) of the global population achieving guideline-target mean intake. There was considerable regional variation, with lowest mean potassium intake reported in Asia, and highest intake in Eastern and Western Europe.

**Supplementary Information:**

The online version contains supplementary material available at 10.1007/s00394-023-03128-6.

## Introduction

Increasing potassium intake, particularly in populations with low potassium intake and high sodium intake, has emerged to be an important population-level intervention to reduce cardiovascular events, based on the findings from cohort studies and randomised controlled trials, including the recently reported large cluster randomised controlled trial, the Salt Substitute and Stroke Study (SSaSS) [1, 2]. In the SSaSS trial, a significant reduction in cardiovascular events and mortality was reported by replacing regular salt (100% Sodium Chloride) with a potassium-enriched salt substitute (75% Sodium Chloride and 25% Potassium Chloride), and resulted in a 57% relative increase in potassium intake and an 8.1% relative reduction in sodium intake [2]. Importantly, the SSaSS trial was conducted in rural China, which is reported to have one of the lowest mean intakes of potassium globally (national mean intake 1.7 g/day), along with one of highest mean intakes of sodium (national mean intake 4.8 g/day) [3, 4], and findings from that trial may only apply to some regions of the world.

Current guidelines recommend a potassium intake of more than 3.5 g/day [5], which is based on clinical trials of blood pressure (BP) lowering, and prospective cohort studies relating potassium intake to mortality and cardiovascular events [1, 6, 7]. Higher potassium intake also appears to diminish the magnitude of association between excess sodium intake and cardiovascular events [8], making potassium intake especially important in populations with high mean sodium intakes.

In this systematic review and meta-analysis, we sought to determine global patterns of potassium intake, along with related sodium intake.

## Methods

We performed a systematic review and meta-analysis, adhering to the Cochrane Collaboration Guidelines and reported our findings according to the Preferred Reporting Items for Systematic Reviews and Meta-Analyses (PRISMA) Guidelines and the Guidelines for Accurate and Transparent Health Reporting (GATHER) statement [9–11]. The meta-analysis was registered with the International Prospective Register of Systematic Reviews (PROSPERO identifier: CRD42022281381). The data that support the findings of this study are available (Supplementary Appendix 2).

### Data sources and search strategy

We systematically searched PubMed and Embase databases from database inception to September 2021. The search terms included are detailed in the Supplementary Appendix (eMethods I). Following removal of duplicates, titles and abstracts were screened by two reviewers (CR and RM) using the Rayann web application [12]. The reference lists of included studies and other published meta-analyses were also reviewed. Full texts of remaining articles were independently assessed by two reviewers (CR and RM), with eligibility based on predetermined criteria (see below). Disagreements were resolved by consensus; where a resolution was not reached by discussion, a consensus was reached through a third reviewer (MOD). The systematic search of articles published before September 2021 identified 7236 records. This search was supplemented with a targeted search of governmental websites and global burden of disease database for data deficient countries (i.e. those that did not have studies which included  > 1000 participants).

### Eligibility criteria

Studies were considered eligible adhered to the following conditions: [1] were representative of a national sample; [2] measured potassium by dietary method (food frequency questionnaire, dietary recall, or food diary) or urinary measurement (24 hour urinary collection, overnight urine, spot or fasting morning urine collection); [3] included greater or equal to 1,000 participants (per country where a sample was collected across multiple countries).

### Data extraction

Data were extracted independently by two authors (CR and AC) using a standardized pre-determined data collection form. For each study, we extracted the title, year of publication, country and region the study was performed in, year of measurement, survey sampling method, population type, representativeness (national or sub-national), method of measurement, formula used where applicable (i.e. if daily intake was formula derived based on spot urine sample), daily mean potassium intake (excretion) and standard deviation. Where reported daily mean sodium intake (excretion) and standard deviation were extracted. Regions were recorded as global burden of disease regions and super-regions [13]. Data were compared for inconsistencies and merged into a pre-final dataset which was checked independently by two other reviewers (RM and VG).

### Outcomes

The primary outcome was mean potassium intake for all age groups, globally, and by region, country and sex. We also report mean potassium intake among adults only (individuals aged  ≥ 18 years old), by region and country. We estimated mean sodium/potassium (Na/K) ratios globally, and by region, country and sex.

### Data standardisation and statistical analysis

Potassium and sodium estimates were standardised to grams per day. The measure of variance was extracted as standard deviation; in studies where standard deviation was not reported, where possible it was derived from available information (e.g., Standard Deviation = Standard Error × square root of sample size) following guidance from the Cochrane handbook [9].

We used dietary questionnaire estimates of potassium intake as the reference standard, since urinary potassium excretion represents 75% of potassium intake [14] (compared to ~ 95% for sodium intake for which we used urinary estimates as reference standard). Where sodium estimates were missing (59 observations [29.8%]), values were imputed for these studies as part of the Markov Chain Monte Carlo (MCMC) procedure [15]. An individual study may correspond to multiple observations, for example if a study reported nationally representative estimates for two countries for both men and women, this study would account for four observations. Potassium means were estimated using a three level Bayesian hierarchical model; according to a study within a country, a country within a region and a region. Global potassium and sodium means were estimated by a population weighted average of the estimated region level means. Within a country it is assumed that study level sodium and potassium mean values are linked via a linear model (eMethods 2). We calculated Na/K ratios derived from both millimole and milligram units, respectively (Na/K and Na/K-mg derived), due to lack of consistency of reporting this metric. Model fitting was performed via Hamiltonian Monte Carlo [15] using RStan [16]. Supplementary appendix 3 contains the source code.

Two additional meta-analyses were performed to estimate the proportion of the population achieving a potassium intake of  > 2.5 g/day and > 3.5 g/day, respectively. Study level proportions achieving these thresholds were estimated by assuming normal distribution within males and females, respectively, based on the estimated mean and standard deviation of potassium intake. The same correction factor used in the primary model for gender and measurement modality was applied to this model. A three-level Bayesian hierarchical model was fit using RStan [16]. The posterior distributions for estimated proportions exceeding each threshold were recovered using a post Markov chain Monte Carlo simulation procedure.

## Results

We identified 193 publications referring to 104 eligible studies; 98 nationally representative surveys and 6 multi-national studies, which corresponded to 198 observations (eFigure 1).

### Study characteristics

104 studies were included which represented data from 52 countries (1,640,664 participants). There was representation of at least one country for 17 of 21 global burden of disease regions [13]. No eligible studies were identified for the following regions: Andean Latin America, Central Asia, Central Sub-Saharan Africa and Oceania (eTable 1). 36 studies reported 24-hour urinary potassium excretion (some of which were formula derived) and 68 reported dietary potassium intake, with 3 studies reporting both urinary and dietary potassium measurements (eTable 2).

### Global potassium intake

The global mean potassium intake was 2.25 g/day [57.59 mmol] (95% credible interval (CI) 2.05–2.44 g/day). The global mean intake was higher in men (2.40 g/day [61.67 mmol] (95% CI 2.20–2.62 g/day)) than women (2.09 g/day [53.51 mmol] (95% CI 1.91–2.27 g/day)) overall and in all regions (Table 1).

**Table 1:** Potassium estimates (g/day) for Country and Region

Region	Country	Overall	Male	Female
		Potassium intake	Potassium intake	Potassium intake
Australasia		2.85 (2.24, 3.47)	3.05 (2.40, 3.72)	2.65 (2.08, 3.23)
	Australia	2.86 (2.24, 3.49)	3.06 (2.39, 3.74)	2.66 (2.08, 3.25)
	New Zealand	2.86 (2.25, 3.49)	3.07 (2.41, 3.74)	2.66 (2.09, 3.24)
Caribbean		2.97 (2.44, 3.50)	3.18 (2.61, 3.75)	2.76 (2.27, 3.25)
	Jamaica	2.94 (2.38, 3.50)	3.15 (2.55, 3.75)	2.73 (2.21, 3.25)
	St. Lucia	3.01 (2.45, 3.58)	3.22 (2.63, 3.84)	2.80 (2.28, 3.33)
	Trinidad and Tobago	2.98 (2.43, 3.52)	3.19 (2.61, 3.77)	2.77 (2.26, 3.27)
Central Europe		2.89 (2.51, 3.26)	3.09 (2.68, 3.50)	2.68 (2.33, 3.03)
	Czech Republic	2.88 (2.48, 3.28)	3.09 (2.65, 3.52)	2.68 (2.30, 3.05)
	Hungary	2.87 (2.42, 3.30)	3.07 (2.59, 3.53)	2.67 (2.25, 3.07)
	Poland	2.93 (2.57, 3.29)	3.13 (2.75, 3.52)	2.72 (2.39, 3.05)
Central Latin America*		2.62 (2.02, 3.23)	2.81 (2.16, 3.46)	2.44 (1.87, 3.00)
	Colombia	2.63 (2.01, 3.24)	2.81 (2.16, 3.48)	2.44 (1.87, 3.01)
	Mexico	2.62 (2.01, 3.23)	2.81 (2.15, 3.47)	2.44 (1.87, 3.01)
East Asia		1.89 (1.55, 2.25)	2.03 (1.66, 2.41)	1.76 (1.44, 2.09)
	China	1.90 (1.64, 2.17)	2.03 (1.75, 2.32)	1.76 (1.52, 2.01)
	Taiwan	1.86 (1.42, 2.24)	1.99 (1.52, 2.41)	1.73 (1.32, 2.08)
Eastern Europe		3.53 (3.05, 4.01)	3.78 (3.26, 4.30)	3.28 (2.83, 3.72)
	Latvia	3.52 (3.00, 4.03)	3.77 (3.21, 4.31)	3.27 (2.79, 3.74)
	Lithuania	3.61 (3.10, 4.16)	3.87 (3.31, 4.46)	3.36 (2.88, 3.87)
	Russia	3.53 (3.02, 4.04)	3.78 (3.23, 4.33)	3.28 (2.80, 3.75)
Eastern Sub Saharan Africa		2.01 (1.22, 2.77)	2.15 (1.31, 2.97)	1.87 (1.14, 2.58)
	Tanzania	1.98 (1.22, 2.73)	2.12 (1.32, 2.93)	1.84 (1.14, 2.54)
High income Asia Pacific		2.41 (2.13, 2.68)	2.58 (2.28, 2.87)	2.24 (1.98, 2.49)
	Japan	2.45 (2.23, 2.67)	2.62 (2.38, 2.86)	2.27 (2.07, 2.48)
	Singapore	2.40 (2.01, 2.74)	2.57 (2.15, 2.94)	2.23 (1.87, 2.55)
	South Korea	2.38 (2.08, 2.66)	2.55 (2.22, 2.85)	2.21 (1.93, 2.47)
High income North America		2.67 (2.27, 3.07)	2.86 (2.43, 3.29)	2.48 (2.11, 2.85)
	Canada	2.71 (2.30, 3.12)	2.90 (2.46, 3.35)	2.51 (2.14, 2.90)
	US	2.64 (2.25, 3.03)	2.82 (2.40, 3.24)	2.45 (2.09, 2.81)
North Africa and Middle East		2.47 (1.98, 2.96)	2.65 (2.12, 3.17)	2.30 (1.85, 2.75)
	Iran	2.48 (1.97, 3.01)	2.66 (2.11, 3.23)	2.31 (1.83, 2.80)
	Palestine	2.45 (1.93, 2.97)	2.62 (2.07, 3.18)	2.28 (1.79, 2.76)
	Turkey	2.48 (1.98, 2.97)	2.65 (2.12, 3.18)	2.30 (1.84, 2.76)
South Asia		1.99 (1.54, 2.46)	2.13 (1.65, 2.64)	1.85 (1.43, 2.29)
	Bangladesh	2.01 (1.52, 2.52)	2.15 (1.63, 2.70)	1.86 (1.41, 2.34)
	India	1.95 (1.54, 2.37)	2.09 (1.65, 2.54)	1.81 (1.43, 2.20)
Southeast Asia		2.00 (1.45, 2.54)	2.15 (1.55, 2.73)	1.86 (1.35, 2.36)
	Malaysia	2.02 (1.45, 2.59)	2.16 (1.55, 2.77)	1.88 (1.34, 2.40)
	Philippines	2.00 (1.41, 2.57)	2.14 (1.52, 2.75)	1.86 (1.31, 2.39)
	Thailand	1.97 (1.38, 2.53)	1.99 (1.52, 2.41)	1.73 (1.32, 2.08)
Southern Latin America		2.23 (1.45, 3.00)	2.39 (1.55, 3.21)	2.07 (1.34, 2.79)
	Argentina	2.22 (1.45, 2.98)	2.37 (1.55, 3.20)	2.06 (1.34, 2.77)
Southern Sub-Saharan Africa		2.06 (1.42, 2.70)	2.21 (1.52, 2.89)	1.92 (1.32, 2.51)
	South Africa	2.04 (1.43, 2.64)	2.19 (1.53, 2.83)	1.90 (1.33, 2.45)
Tropical Latin America		2.62 (2.06, 3.17)	2.80 (2.21, 3.40)	2.43 (1.91, 2.94)
	Brazil	2.62 (2.11, 3.14)	2.80 (2.26, 3.36)	2.44 (1.96, 2.92)
Western Europe		3.30 (3.13, 3.47)	3.53 (3.34, 3.73)	3.07 (2.91, 3.22)
	Austria	3.27 (2.94, 3.55)	3.50 (3.14, 3.81)	3.04 (2.73, 3.30)
	Belgium	3.29 (3.00, 3.56)	3.52 (3.21, 3.82)	3.06 (2.79, 3.31)
	Denmark	3.31 (3.03, 3.60)	3.54 (3.24, 3.86)	3.07 (2.82, 3.34)
	Estonia	3.51 (2.99, 4.02)	3.76 (3.20, 4.31)	3.26 (2.78, 3.74)
	Finland	3.33 (3.05, 3.68)	3.57 (3.26, 3.94)	3.10 (2.83, 3.42)
	France	3.22 (2.91, 3.45)	3.45 (3.12, 3.71)	2.99 (2.71, 3.21)
	Germany	3.24 (2.90, 3.48)	3.47 (3.10, 3.74)	3.01 (2.70, 3.24)
	Greece	3.25 (2.92, 3.51)	3.48 (3.12, 3.77)	3.02 (2.72, 3.26)
	Ireland	3.29 (2.99, 3.57)	3.52 (3.20, 3.83)	3.06 (2.78, 3.31)
	Italy	3.33 (3.09, 3.59)	3.56 (3.30, 3.85)	3.09 (2.88, 3.34)
	Netherlands	3.39 (3.14, 3.72)	3.63 (3.36, 3.99)	3.15 (2.92, 3.46)
	Norway	3.34 (3.07, 3.66)	3.58 (3.29, 3.92)	3.10 (2.86, 3.40)
	Portugal	3.33 (3.04, 3.66)	3.57 (3.25, 3.93)	3.09 (2.83, 3.40)
	Spain	3.31 (3.06, 3.57)	3.55 (3.27, 3.83)	3.08 (2.85, 3.32)
	Sweden	3.28 (2.98, 3.55)	3.52 (3.19, 3.81)	3.05 (2.77, 3.30)
	Switzerland	3.27 (2.94, 3.54)	3.50 (3.15, 3.80)	3.04 (2.74, 3.29)
	UK	3.37 (3.16, 3.64)	3.61 (3.37, 3.90)	3.13 (2.94, 3.38)
Western Sub Saharan Africa		1.92 (1.42, 2.43)	2.06 (1.52, 2.61)	1.79 (1.32, 2.26)
	Cameroon	1.91 (1.37, 2.45)	2.04 (1.47, 2.62)	1.77 (1.28, 2.27)
	Ghana	1.96 (1.42, 2.51)	2.10 (1.53, 2.70)	1.82 (1.32, 2.37)
	Nigeria	1.88 (1.37, 2.38)	2.01 (1.46, 2.55)	1.74 (1.27, 2.21)

Mean (95% credible intervals)

*Estimates including adults only: Central Latin America- 2.96 (2.33, 3.60), Columbia-2.95 (2.30, 3.59), Mexico-3.00 (2.35, 3.66)

### Regional potassium intake

Table 1 reports regional and country-level mean potassium intake. There were regional differences in mean potassium intake, with the lowest mean potassium intake reported in East Asia, 1.89g/day (95% CI 1.55–2.25 mg/day), and the highest mean potassium intake was reported in Eastern Europe at 3.53 g/day (95% CI 3.05–4.01 g/day).

Approximately 35% (95% CI 30–41%) of population of regions included (which excluded 5.5% of the world’s population due to lack of data (eTable 2)) had an estimated potassium intake  > 2.5 g/day. 14% (95% CI 11–17%) of population included reported a mean potassium above 3.5 g/day (i.e., an intake meeting WHO recommended intake).

**Fig. 1:**
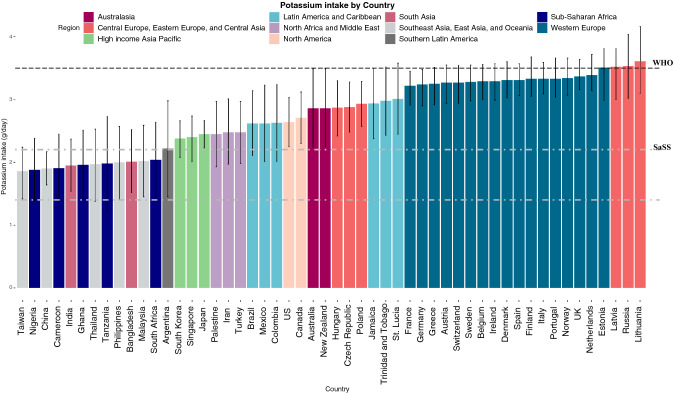
Potassium estimates by country and region ranked by potassium intake (g/day). Bar chart depicting mean potassium intake by country and region. The y-axis represents potassium intake in grams per day (g/day), ordered by mean potassium intake from lowest daily intake to highest daily intake. Error bars represent 95% credible intervals. The x-axis depicts countries included. The bar colour represents regions as described in the legend; light grey- South East Asia, East Asia and Oceania, navy- Sub-Saharan Africa, pink- South Asia, dark grey- Southern Latin America, green- High income Asia Pacific, purple- North Africa and Middle East, blue- Latin America and Caribbean, peach- North America, dark red- Australasia, coral- Central/Eastern Europe and Central Asia, turquoise- Western Europe. The dashed black line represents the lower limit of WHO potassium recommendation of 3.5 g. The dashed white lines represent potassium intake reported in the SSaSS, the lower line being prior to intervention (1.4 g) and upper line being post intervention (2.2 g)

**Fig. 2:**
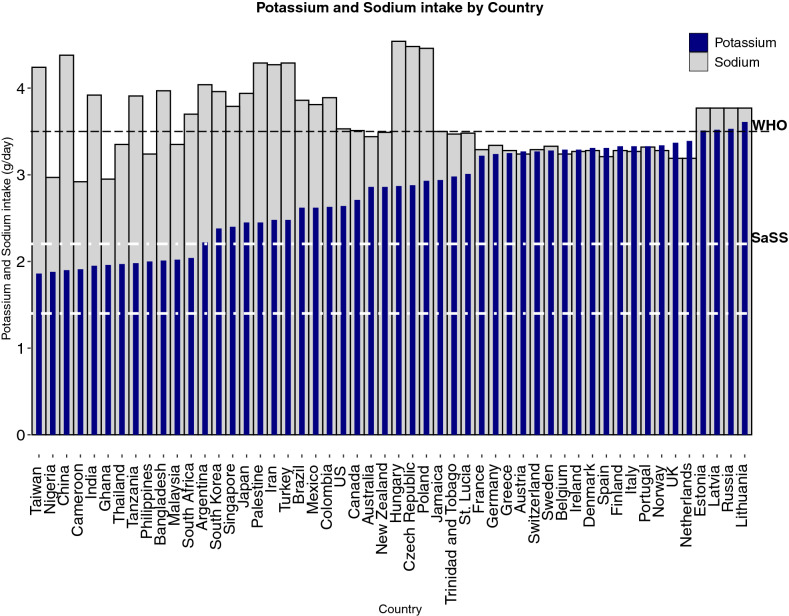
Potassium and sodium estimates by country ranked by potassium intake (g/day). Bar chart depicting mean potassium and sodium intakes respectively by country. The navy column represents potassium intake and the grey column represents sodium intake. The y-axis represents intake in grams per day (g/day). The x-axis depicts countries included. The dashed black line represents the lower limit of WHO potassium recommendation of 3.5 g/day. The dashed white lines represent potassium intake reported in the SSaSS, the lower line being prior to intervention (1.4 g/day) and upper line being post intervention (2.2 g/day)

Estimated mean potassium intake among six regions (East Asia, Western Sub-Saharan Africa, South Asia, South-east Asia, Eastern Sub-Saharan Africa, Southern Sub-Saharan Africa) reported mean potassium intake lower than that achieved post intervention in the SaSS trial (i.e. lower than 2.20 g/day) with intakes ranging from 1.89 mg (95% CI 1.55–2.25 g) in East Asia and 2.06 g (95% CI 1.42–2.70 g) in Southern Sub-Saharan Africa (Figs. 1, and 2).

### Country-level potassium intake

Among individual countries, the highest potassium intake was reported in Lithuania, with lowest intake reported in Taiwan (Table 1, Fig. 1). Four countries reported mean potassium intake of  > 3.5 g; Estonia, Latvia, Russia and Lithuania. eTable 3 reports regional and country level intake in an adult population only which did not materially alter our finding.

### Na/K ratio

**Fig. 3:**
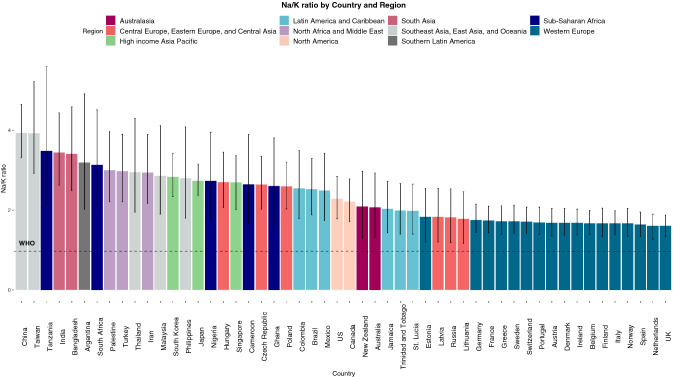
Sodium/Potassium ratio estimates (mmol derived) by Country and Region, ranked by Sodium/Potassium ratio. Bar chart depicting Na/K ratio by country and region. The bar colour represents regions as described in the legend; light grey- South East Asia, East Asia and Oceania, navy- Sub-Saharan Africa, pink- South Asia, dark grey-Southern Latin America, green- High income Asia Pacific, purple- North Africa and Middle East, blue- Latin America and Caribbean, peach- North America, dark red- Australasia, coral- Central/Eastern Europe and Central Asia, turquoise- Western Europe. The y-axis represents Na/K ratio (mmol derived), ordered by Na/K ratio from highest to lowest. Error bars represent 95% credible intervals. The dashed black line represents the recommended ratio for sodium/potassium (1.0) as per WHO guidelines

The global mean mmol Na/K ratio was 2.88 (95% CI 2.56–3.23). The highest regional Na/K ratios were reported in East Asia (3.85; 95% CI 3.04–4.81), Eastern Sub-Saharan Africa (3.42; 95% CI 2.12–5.52) and South Asia (3.39; 95% CI 2.54–4.45) regions. The lowest Na/K ratios were reported in Western Europe (1.68; 95% CI 1.46–1.92) and Eastern Europe (1.82; 95% CI 1.23–2.48) regions (eTable 4, Fig. 3).

Among individual countries, the highest mmol Na/K ratio was reported in China (3.93; 95% CI 3.31–4.64), with the lowest Na/K ratio reported in the Netherlands (1.60; 95% CI, 1.27-1.90) and the United Kingdom (1.60; 95% CI 1.33–1.87) (eTable 4, Fig. 3, eFigure 2). Na/K mg derived ratio are reported in eTable 5 and represented in eFigures 3a and b.

### World health organisation targets

**Fig. 4:**
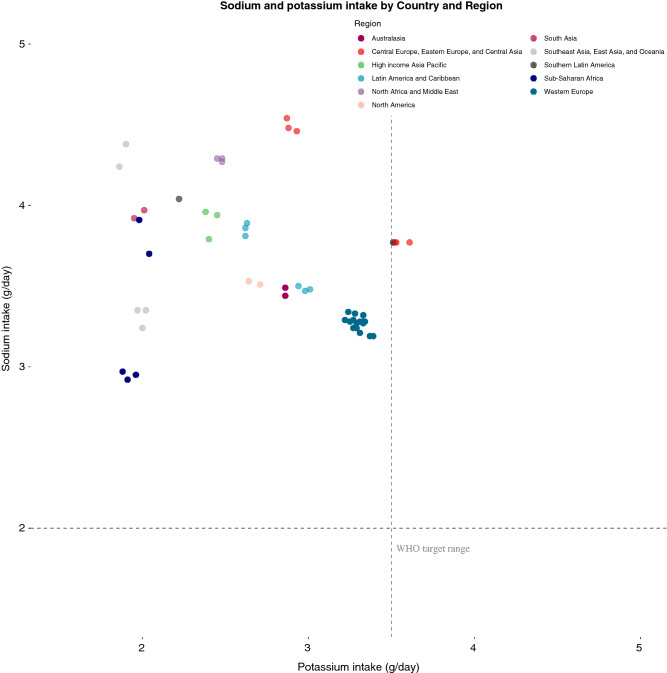
Sodium and Potassium estimates by Country and Region. Plot depicting sodium and potassium estimates by country and region. Each dot represents a country estimate. Countries are plotted based on mean potassium and mean sodium estimates, respectively. The colour represents regions as described in the legend; light grey- South East Asia, East Asia and Oceania, navy- Sub-Saharan Africa, pink- South Asia, dark grey- Southern Latin America, green- High income Asia Pacific, purple- North Africa and Middle East, blue- Latin America and Caribbean, peach- North America, dark red- Australasia, coral- Central/Eastern Europe and Central Asia, turquoise- Western Europe. The dashed lines represents the recommended intake of sodium and potassium, respectively, as per WHO guidelines

No country-level study reported combined intakes of sodium and potassium that achieved World Health Organisation targets, i.e. potassium intake of  > 3.5 g/day, while reducing sodium intake to  < 2 g/day (Figs. 2 and 4).

## Discussion

Our systematic review and meta-analysis reports an estimated global mean daily potassium intake (from available country-level data sources), as well as intake by region, country and gender. The World Health Organisation recommends a target potassium intake of  > 3.5 g/day, while reducing sodium intake to  < 2 g/day [5, 17], a combined recommendation achieved by none of the countries included in our review. We found that current potassium intake targets are not being achieved at global population level (estimated global potassium intake of 2.25 g/day), regional level (with the possible exception of Eastern Europe) or country level (with the exception of four out of fifty-two included countries). We noted marked regional differences, with lowest potassium intake reported in Asia and Africa, although given limited data availability for the latter there is uncertainty around this estimate. We report that no region achieves the recommended Na/K ratio of 1.0, with the lowest Na/K reported in Western Europe and highest in East Asia [18].

Our study provides a summary meta-analytic estimate of potassium intake globally. Prior studies, including a narrative review by Van Mierlo et al, reported national mean intakes ranging from 1.7 to 3.7 g/day among 21 countries, with lowest intake reported in China [3]. Within individual international studies (e.g. INTERSALT and PURE studies), similar regional variation is reported, with higher potassium excretion in European countries compared to Asia and Africa [19, 20]. We report mean potassium intake within major regions of the world which provides key information on which regions, and countries, might be most suited to the salt substitute employed in the SSaSS trial. However, widespread use of the salt substitute would also need to be guided by knowledge of whether most sodium intake is derived from discretionary or non-discretionary sources, since advocating salt substitutes in communities where most sodium is derived from non-discretionary sources might result in paradoxically increasing sodium intake.

Potassium is an essential mineral and current guideline recommended adequate intakes are based on evidence from blood pressure lowering effects [1], and prospective cohort studies evaluating the association of potassium intake and incidence of cardiovascular events and stroke [21–23]. However, the optimal intake range is uncertain, reflected in variations in the adequate intake range between guideline bodies, for example the National Academy of Medicine define adequate intake as 3400 mg for men and 2600 mg for women (which is a reduction from 2005 guideline (4700 mg for both men and women), following a change to methodology of defining adequate intake)), and the European Food and Safety Authority set adequate intake as 3500 mg for both sexes [6, 24]. Sub-optimal potassium intake has led to policy changes in some countries, for example regulations in the United States passed in 2016 mandate displaying potassium content of food on the label.

Although population-level potassium intake has received less attention than sodium intake as a public health strategy to reduce cardiovascular risk, the results of the recent SSaSS trial might suggest that increasing potassium intake may be of greater importance to public health strategies [2, 26]. This study achieved a 57% (803 mg) increase in potassium and 8.1% (350 mg) reduction in sodium resulting in 3.34 mmHg reduction in systolic blood pressure, through salt substitution with 25% KCl, without otherwise modifying dietary quality [2]. A previous cluster randomised trial conducted in Taiwan which randomised clusters to potassium enriched salt also found a significant reduction in cardiovascular mortality in the experimental group, also reporting larger changes in potassium intake than sodium intake [27]. Sodium and potassium intakes are correlated in general populations and their association with cardiovascular events and mortality is interdependent [8]; therefore, strategies to optimise intake should consider both nutrients. Some guidelines make joint recommendations advising  < 2g of sodium and  > 3.5g of potassium; however, observational studies demonstrate that less than 1% of the population achieve this joint target, and this joint target was not achieved in any country included in our review [6, 8, 28]. The application of the findings of the SSaSS trial is dependent on identifying regions of the world which are potassium deplete (we note that approximately two-thirds of the population have a potassium intake  < 2.5 g), with a high sodium intake. Implementation of strategies to increase potassium intake must also be underpinned by an understanding of sources of sodium intake, with countries where discretionary sodium intake is high being appropriate targets for salt supplementation strategies. Previous studies estimate that discretionary sodium intake accounts for more than half of sodium intake in South East Asia; in contrast, it is estimated that the majority of sodium intake in the United States is outside the home, e.g. during food processing with approximately 10% of sodium intake accounted for in the home (i.e., added at the table, or during cooking) [29, 30]. As outlined in the WHO salt reduction strategy, interventions should be tailored to individual countries; discretionary salt intake and cooking practices. Encouraging adherence with dietary guideline recommendations to increase intake of foods items that are rich in potassium (fruit, vegetables and nuts) is an obvious approach to population-level prevention of cardiovascular events. There are challenges to achieving a potassium replete diet such as affordability and food availability. Previous analyses of diets such as the EAT-Lancet diet estimated that the cost exceeded the household per capita income for 1.58 billion people [32]. These observations invite the question of whether potassium supplements, independent of salt substitutes, should be evaluated for cardiovascular prevention in populations.

The Global Burden of Diseases reported mean intakes of sodium in different regions of the world, reporting a global sodium intake above recommended range at 3.95 g/day [4]. Our analytic approach differs from the Global Burden of Disease meta-analytic approach in several ways. First, where there were no data available at country-level we have not reported a country-level estimate, rather a region estimate based on available data. In contrast, prior Global Burden of Disease studies for example the systematic analysis of sodium intakes by Powles et al. [4], impute data for data-deficient countries using information from neighbourring countries. Second, we have applied a correction factor within the model to convert urinary potassium estimates to dietary estimates (which were on average 21% (95% CI 4–30%) higher than urinary potassium estimates), where the Powles et al. study applied an additional pre-processing step to standardise sodium data by cross-walk validation [4]. Given the low number of studies with paired urinary and dietary potassium data, we did not apply this approach. An advantage of our approach, which included the correction factor within the model, is that variability in the estimated conversions were propagated automatically into the variability of the final estimates. Finally, our primary model was a joint model for potassium and sodium, in contrast to Powles et al. meta-analytic estimate of global sodium intake which modelled this electrolyte alone.

Given that sodium and potassium intake are correlated, and interdependent, the proposed use of a sodium/potassium ratio has received attention. However, there are several challenges with use of this metric, including lack of consensus regarding measurement units. For example, use of millimole versus milligram units will provide different ratio values, necessitating work on a standardized approach to reporting. There are difficulties in interpreting the healthiness of an individual diet based on a given ratio, for example diets low in potassium and sodium may render a ‘recommended’ ratio but may be poor in intake of fruits, vegetables and nuts.

### Limitations of our study

Our study has several potential limitations. First, despite an extensive search, including a systematic search and targeted search of governmental websites for data-deficient countries, there were limited data available for regions of the world, e.g. Sub-Saharan Africa. Therefore, estimates of global intake must be interpreted with caution given that they are imputed based on the limited data available. Our results highlight data gaps in nutritional assessment including measurement of nutrients such as potassium, particularly in lower income countries. Second, there was variation in the year of measurement with sampling ranging from 1985 to 2020. However, evidence suggests that there has been a reduction in potassium intake over time [33]; therefore, inclusion of surveys from earlier time points are likely to over-estimate current potassium intake. Third, there was variation in method of measurement. All methods of measurement were included to maximise the number of countries represented with a correction factor utilised to standardise variability related to measurement method. However, our results are consistent with findings of studies which used repeated 24-hour urinary measurements. In an individual meta-analysis of six prospective cohort studies which completed repeated 24 hour urinary collections (which included participants from the United States and the Netherlands), Ma et al. also reported potassium intakes below the recommended range, reporting a median potassium excretion of 2535 mg [34]. Fourth, we report estimates overall and among adults only; we do not report estimates by age group. Last, we did not systematically search for studies estimating sodium intake; therefore, sodium estimates should be interpreted with caution. The primary aim of our study was to estimate potassium intake; therefore, our search strategy aimed to identify studies which reported daily potassium intake of a nationally representative sample, where sodium excretion (intake) was reported this data was extracted.

## Conclusions

Global mean potassium intake (2.25 g/day) falls below current guideline recommended intake level of  > 3.5g/day [17], with only 35% of the global population estimated to achieve this target. There is considerable regional variation, with lowest mean potassium intake reported in Asia, and highest intake in Eastern and Western Europe.

## Supplementary Information

Below is the link to the electronic supplementary material.

Supplementary file1 (DOCX 22 KB)

Supplementary file2 (XLSX 46 KB)

Supplementary file3 (DOCX 400 KB)

## Data Availability

All data generated or analysed during this study are included in this published article (and its supplementary information files).

## Abbreviations

SSaSS: Salt substitute and stroke study
NaCl: Sodium chloride
KCl: Potassium chloride
CI: Credible interval
Na/K: Sodium/potassium
