# Supplementary material for: Global mean potassium intake: a systematic review and Bayesian meta-analysis
Source: Eur J Nutr. 2023 Mar 8;62(5):2027–37. doi: 10.1007/s00394-023-03128-6 (PMC10349712; doi:10.1007/s00394-023-03128-6)
Supplement: Supplementary file 1 — Supplementary file1 (DOCX 22 KB) [file 394_2023_3128_MOESM1_ESM.docx]

#Clear the environment

rm(list = setdiff(ls(), lsf.str()))

while (dev.cur()>1) dev.off()

#Load libraries

library(dplyr)

library(reshape2)

library("rstan")

#Load data

#”Potassium_Data_24_03_2022.Rdata"

dat$Gender[dat$Percentage_female==100] <- "Female"

dat$Gender[is.na(dat$Mean_potassium) & (is.na(dat$Percentage_female) | (dat$Percentage_female<100))] <- 'Both'

dat$Country[dat$Country=="The Netherlands"] <- "Netherlands"

dat$Mean_sodium[is.na(dat$Mean_sodium)] <- dat$Mean_sodium_total[is.na(dat$Mean_sodium)]

dat$Mean_potassium[is.na(dat$Mean_potassium)] <- dat$Mean_potassium_total[is.na(dat$Mean_potassium)]

## remove rows when misassigned gender, when study is an overall study

dat <- dat[-(1:nrow(dat))[dat$studyID %in% unique(dat$studyID[dat$Gender=='Both']) & dat$Gender!="Both"],]

### remove duplicate rows

dat <- dat[!duplicated(paste0(dat$studyID,dat$Gender)),]

dat <- dat[!is.na(dat$Region),]

N <- nrow(dat)

J <- length(unique(dat$studyID))

C <- length(unique(dat$Country))

R <- length(unique(dat$Region))

# code below will only work if data is ordered recursively by studyid within region within country

dat <- dat[order(dat$Region,dat$Country,dat$studyID),]

id <- rep(1,length(dat$studyID))

for(i in 2:length(id)){

id[i] <- id[i-1]

if(dat$studyID[i]!=dat$studyID[i-1]) id[i] <- id[i]+1

}

country <- rep(1,length(dat$Country))

for(i in 2:length(id)){

country[i] <- country[i-1]

if(dat$Country[i]!=dat$Country[i-1]) country[i] <- country[i]+1

}

region <- rep(1,length(dat$Region))

for(i in 2:length(id)){

region[i] <- region[i-1]

if(dat$Region[i]!=dat$Region[i-1]) region[i] <- region[i]+1

}

region_country <- rep(1,length(unique(dat$Country)))

dat_2 <- dat[!duplicated(dat$Country),]

for(i in 2:length(region_country)){

region_country[i] <- region_country[i-1]

if(dat_2$Region[i]!=dat_2$Region[i-1]) region_country[i] <- region_country[i]+1

}

region_pop <- dat$Popu[!duplicated(dat$Region)]

## need to replace by correct standard errors for sodium and potassium

SE_P <- mean(dat$SD_potassium_total,na.rm=TRUE)/sqrt(dat$SampleSize)

SE_S <- mean(dat$SD_sodium_total,na.rm=TRUE)/sqrt(dat$SampleSize)

dat$Percentage_female[is.na(dat$Percentage_female)] <- 50

SE_P[dat$Gender=='Female'] <- mean(dat$SD_potassium_total,na.rm=TRUE)/sqrt(dat$SampleSize[dat$Gender=='Female']*dat$Percentage_female[dat$Gender=='Female']/100)

SE_S[dat$Gender=='Female'] <- mean(dat$SD_sodium_total,na.rm=TRUE)/sqrt(dat$SampleSize[dat$Gender=='Female']*dat$Percentage_female[dat$Gender=='Female']/100)

SE_P[dat$Gender=='Male'] <- mean(dat$SD_potassium_total,na.rm=TRUE)/sqrt(dat$SampleSize[dat$Gender=='Male']*(100-dat$Percentage_female[dat$Gender=='Male'])/100)

SE_S[dat$Gender=='Male'] <- mean(dat$SD_sodium_total,na.rm=TRUE)/sqrt(dat$SampleSize[dat$Gender=='Male']*(100-dat$Percentage_female[dat$Gender=='Male'])/100)

SE_P[dat$Gender=='Female' &!is.na(dat$SD_potassium_total)] <- dat$SD_potassium_total[dat$Gender=='Female' &!is.na(dat$SD_potassium_total)]/sqrt(dat$SampleSize[dat$Gender=='Female' &!is.na(dat$SD_potassium_total)]*dat$Percentage_female[dat$Gender=='Female' &!is.na(dat$SD_potassium_total)]/100)

SE_S[dat$Gender=='Female' &!is.na(dat$SD_sodium_total)] <- dat$SD_sodium_total[dat$Gender=='Female' &!is.na(dat$SD_sodium_total)]/sqrt(dat$SampleSize[dat$Gender=='Female' &!is.na(dat$SD_sodium_total)]*dat$Percentage_female[dat$Gender=='Female' &!is.na(dat$SD_sodium_total)]/100)

SE_P[dat$Gender=='Male' &!is.na(dat$SD_potassium_total)] <- dat$SD_potassium_total[dat$Gender=='Male' &!is.na(dat$SD_potassium_total)]/sqrt(dat$SampleSize[dat$Gender=='Male' &!is.na(dat$SD_potassium_total)]*(100-dat$Percentage_female[dat$Gender=='Male' &!is.na(dat$SD_potassium_total)])/100)

SE_S[dat$Gender=='Male' &!is.na(dat$SD_sodium_total)] <- dat$SD_sodium_total[dat$Gender=='Male' &!is.na(dat$SD_sodium_total)]/sqrt(dat$SampleSize[dat$Gender=='Male' &!is.na(dat$SD_sodium_total)]*(100-dat$Percentage_female[dat$Gender=='Male' &!is.na(dat$SD_sodium_total)])/100)

SE_P[dat$Gender=='Both' &!is.na(dat$SD_potassium_total)] <- dat$SD_potassium_total[dat$Gender=='Both' &!is.na(dat$SD_potassium_total)]/sqrt(dat$SampleSize[dat$Gender=='Both' &!is.na(dat$SD_potassium_total)])

SE_S[dat$Gender=='Both' &!is.na(dat$SD_sodium_total)] <- dat$SD_sodium_total[dat$Gender=='Both' &!is.na(dat$SD_sodium_total)]/sqrt(dat$SampleSize[dat$Gender=='Both' &!is.na(dat$SD_sodium_total)])

dat$Percentage_female[dat$Gender=="Female"] = 100

dat$Percentage_female[dat$Gender=="Male"] = 0

dat$GDP[dat$Country=='Taiwan'] <- 32787

dietary <- as.numeric(dat$Dietary_or_urine_method=='Dietary')

dietary[is.na(dietary)] <- 1 # assume that study was dietary when this variable is missing... (Just one study)

pot <- dat$Mean_potassium

sod <- dat$Mean_sodium

gdp <- log(dat$GDP[!duplicated(dat$Country)]) # use log GDP

gdp2 <- gdp^2

ii_miss <- (1:nrow(dat))[is.na(dat$Mean_sodium)]

ii_obs <- setdiff((1:nrow(dat)),ii_miss)

N_obs <- length(ii_obs)

N_miss <- length(ii_miss)

sod_obs <- sod[ii_obs]

gender=(100-dat$Percentage_female)/100

options(mc.cores=parallel::detectCores())

scode <- "data{

int<lower=0> N; // number data points

int<lower=0> N_obs; // number fully observed data points

int<lower=0> N_miss; //number missing data points

int<lower=1, upper=N> ii_obs[N_obs];

int<lower=1, upper=N> ii_miss[N_miss];

int<lower=0> J; // number studies

int<lower=0> C; // number countries

int<lower=0> R; // number of regions

real<lower=0> SE_S[N]; // sodium standard error

real<lower=0> SE_P[N]; // potassium standard error

int<lower=0> id[N]; // study id

int<lower=0> country[N]; //country id

int<lower=0> region[N]; //region id

int<lower=0> region_country[C]; //region id going over list of countries

real<lower=0> gender[N]; //gender (0 for females, .5 for both and 1 for males)

real<lower=0> pot[N]; //average potassium

real<lower=0> sod_obs[N_obs]; //observed sodium on non missing indexes

int<lower=0> dietary[N]; //indicator for dietary study

}

parameters{

real<lower=0,upper=3339> mu_potassium; //worldwide mean potassium

real<lower=0,upper=678> tau_study; //between study sd for potassium

real<lower=0,upper=646> tau_country; //between country sd for potassium

real<lower=0,upper=2248> tau_region; //between region sd for potassium

real<lower=0,upper=500> tau_gender; //sd for gender effect on potassium between studies

real<lower=0, upper= 3030> tau_beta0_region; //between region sd for potassium sodium intercept

real<lower=0,upper=1.53> tau_beta1_region; //between region sd for potassium sodium slope

real<lower=0, upper= 3030> tau_beta0_country; //between country sd for potassium sodium intercept

real<lower=0,upper=1.53> tau_beta1_country; //between country sd for potassium sodium slope

real<lower=0,upper=1527> sigma_study; //between study sd for sodium

real<lower=0,upper=500> sigma_gender; //sd for gender effect on sodium between studies

real<lower=0,upper=7000> beta_0; //average intecept for relationship between potassium (x) and sodium (y)

real<lower=-1.68,upper=1.68> beta_1; //between slope for relationship between potassium (x) and sodium (y)

real<lower=-0.5, upper=0.5> R_sodium_diet; //ratio: dietary measurment/urinary measurment for sodium

real<lower=-0.5, upper=0.5> R_potassium_diet; //ratio: dietary measurment/urinary measurment for potassium

real<lower=0, upper=1> R_gender_potassium;// difference between male and female potassium

real<lower=0, upper=1> R_gender_sodium;// difference between male and female sodium

vector[J] eta_study_potassium; //study level random effects

vector[C] eta_country_potassium; //country level random effects

vector[R] eta_region_potassium; //region level random effects

vector[J] eta_study_sodium; //study level random effects

vector[C] eta_country_sodium; //country level random effects

vector[R] eta_0_region; //region level random effects for intercept

vector[R] eta_1_region; //region level random effects for slope

vector[C] eta_0_country; //country level random effects for intercept

vector[C] eta_1_country; //country level random effects for slope

vector[J] eta_gender_potassium; //study level deviations of potassium gender effect from overall gender effect potassium

vector[J] eta_gender_sodium; //study level deviations of potassium gender effect from overall gender effect for sodium

real<lower=1000, upper=6500> sod_miss[N_miss];

}

transformed parameters{

vector[R] beta_1_region; // region level random slopes

vector[R] beta_0_region; // region level random intercepts

vector[C] beta_1_country; // country level random slopes

vector[C] beta_0_country; // country level random intercepts

vector[N] theta_potassium; // mean potassium within study

vector[N] theta_potassium_raw; // mean potassium within study, if female and urinary measurment

vector[C] theta_country_potassium; //country level effects

vector[R] theta_region_potassium; //region level effects

vector[R] theta_region_sodium; //region level effects

vector[N] theta_sodium; // mean potassium within study

vector[C] theta_country_sodium; //country level effects for sodium (based on potassium)

real sod[N];

sod[ii_obs] = sod_obs;

sod[ii_miss] = sod_miss;

for(i in 1:R){

beta_0_region[i] = beta_0 + eta_0_region[i]*tau_beta0_region;

beta_1_region[i] = beta_1 + eta_1_region[i]*tau_beta1_region;

theta_region_potassium[i] = mu_potassium + eta_region_potassium[i]*tau_region;

theta_region_sodium[i] = beta_0_region[i];

}

for(i in 1:C){

beta_0_country[i] = beta_0_region[region_country[i]] + eta_0_country[i]*tau_beta0_country;

beta_1_country[i] = beta_1_region[region_country[i]] + eta_1_country[i]*tau_beta1_country;

theta_country_potassium[i] = mu_potassium + eta_country_potassium[i]*tau_country + eta_region_potassium[region_country[i]]*tau_region;

theta_country_sodium[i] = beta_0_country[i];

}

for(i in 1:N){

theta_potassium_raw[i] = mu_potassium + eta_study_potassium[id[i]]*tau_study + eta_country_potassium[country[i]]*tau_country + eta_region_potassium[region[i]]*tau_region;

theta_potassium[i] = ((mu_potassium + eta_study_potassium[id[i]]*tau_study + eta_country_potassium[country[i]]*tau_country + eta_region_potassium[region[i]]*tau_region)*(1+ R_gender_potassium*gender[i]) + eta_gender_potassium[id[i]]*tau_gender*gender[i])*(1+R_potassium_diet*dietary[i]);

theta_sodium[i] = ((eta_study_sodium[id[i]]*sigma_study + beta_0_country[country[i]] + beta_1_country[country[i]]*(theta_potassium_raw[i]-theta_country_potassium[country[i]]))*(1+R_gender_sodium*gender[i]) + eta_gender_sodium[id[i]]*sigma_gender*gender[i])*(1+R_sodium_diet*dietary[i]);

}

}

model{

eta_0_region ~ normal(0,1);

eta_1_region ~ normal(0,1);

eta_0_country ~ normal(0,1);

eta_1_country ~ normal(0,1);

eta_study_potassium ~ normal(0,1);

eta_country_potassium ~ normal(0,1);

eta_region_potassium ~ normal(0,1);

eta_gender_potassium ~ normal(0,1);

pot ~ normal(theta_potassium, SE_P);

eta_study_sodium ~ normal(0,1);

eta_country_sodium ~ normal(0,1);

eta_gender_sodium ~ normal(0,1);

sod ~ normal(theta_sodium, SE_S);

}"

writeLines(scode,"joint_3Mar.stan")

joint_fit <- stan(file="joint_3Mar.stan",data=c("N","N_obs","N_miss","ii_obs","ii_miss","J","C","R","SE_S","SE_P","id","country","region","gender","region_country","pot","sod_obs","dietary"),iter=10000,chains=8,control = list(max_treedepth = 15))

options(max.print=10^8)

sink("joint_24Mar_summary.txt")

print(joint_fit)

sink()

joint_sim <- extract(joint_fit, pars=c("mu_potassium","tau_study","tau_country","tau_region","tau_gender","tau_beta0_region","tau_beta1_region","tau_beta0_country","tau_beta1_country","sigma_study","sigma_gender","beta_0","beta_1","beta_0_region","beta_1_region","beta_0_country","beta_1_country","R_sodium_diet","R_potassium_diet","R_gender_sodium","R_gender_potassium","theta_country_potassium","theta_region_potassium","theta_country_sodium","theta_region_sodium"),include=TRUE)

## seems to be well converged.

save("joint_sim","dat",file="joint_sim_24Mar_MCMC")

## rescale so that country and region level sodium and potassium are for average of males and females (also put potassium on dietary scale)

joint_sim$theta_country_potassium <- joint_sim$theta_country_potassium*(1 + matrix(rep(joint_sim$R_gender_potassium,ncol(joint_sim$theta_country_potassium)),ncol=ncol(joint_sim$theta_country_potassium))/2)*(1 + matrix(rep(joint_sim$R_potassium_diet,ncol(joint_sim$theta_country_potassium)),ncol=ncol(joint_sim$theta_country_potassium)))

joint_sim$theta_country_sodium <- joint_sim$theta_country_sodium*(1 + matrix(rep(joint_sim$R_gender_sodium,ncol(joint_sim$theta_country_sodium)),ncol=ncol(joint_sim$theta_country_sodium))/2)

joint_sim$theta_region_potassium <- joint_sim$theta_region_potassium*(1 + matrix(rep(joint_sim$R_gender_potassium,ncol(joint_sim$theta_region_potassium)),ncol=ncol(joint_sim$theta_region_potassium))/2)*(1 + matrix(rep(joint_sim$R_potassium_diet,ncol(joint_sim$theta_region_potassium)),ncol=ncol(joint_sim$theta_region_potassium)))

joint_sim$theta_region_sodium <- joint_sim$theta_region_sodium*(1 + matrix(rep(joint_sim$R_gender_sodium,ncol(joint_sim$theta_region_sodium)),ncol=ncol(joint_sim$theta_region_sodium))/2)

## calculate world wide mean and region level means by gender....

joint_sim$world_wide_mean_potassium <- apply(joint_sim$theta_region_potassium,1,function(x){weighted.mean(x,w=region_pop)})

joint_sim$world_wide_mean_sodium <- apply(joint_sim$theta_region_sodium,1,function(x){weighted.mean(x,w=region_pop)})

joint_sim$world_wide_mean_potassium_male <- joint_sim$world_wide_mean_potassium*(1+joint_sim$R_gender_potassium)/(1+joint_sim$R_gender_potassium/2)

joint_sim$world_wide_mean_sodium_male <- joint_sim$world_wide_mean_sodium*(1+joint_sim$R_gender_sodium)/(1+joint_sim$R_gender_sodium/2)

joint_sim$world_wide_mean_potassium_female <- joint_sim$world_wide_mean_potassium/(1+joint_sim$R_gender_potassium/2)

joint_sim$world_wide_mean_sodium_female <- joint_sim$world_wide_mean_sodium/(1+rep(joint_sim$R_gender_sodium)/2)

## world wide ratios

joint_sim$world_wide_ratio_mg <- joint_sim$world_wide_mean_sodium/joint_sim$world_wide_mean_potassium

joint_sim$world_wide_ratio_female_mg <- joint_sim$world_wide_mean_sodium_female/joint_sim$world_wide_mean_potassium_female

joint_sim$world_wide_ratio_male_mg <- joint_sim$world_wide_mean_sodium_male/joint_sim$world_wide_mean_potassium_male

joint_sim$world_wide_ratio_moles <- joint_sim$world_wide_ratio_mg*(39/23)

joint_sim$world_wide_ratio_female_moles <- joint_sim$world_wide_ratio_female_mg*(39/23)

joint_sim$world_wide_ratio_male_moles <- joint_sim$world_wide_ratio_male_mg*(39/23)

joint_sim$theta_region_sodium_male <- joint_sim$theta_region_sodium*matrix(1+rep(joint_sim$R_gender_sodium,17),ncol=17)/matrix(1+rep(joint_sim$R_gender_sodium,17)/2,ncol=17)

joint_sim$theta_country_sodium_male <- joint_sim$theta_country_sodium*matrix(1+rep(joint_sim$R_gender_sodium,52),ncol=52)/matrix(1+rep(joint_sim$R_gender_sodium,52)/2,ncol=52)

joint_sim$theta_region_potassium_male <- joint_sim$theta_region_potassium*matrix(1+rep(joint_sim$R_gender_potassium,17),ncol=17)/matrix(1+rep(joint_sim$R_gender_potassium,17)/2,ncol=17)

joint_sim$theta_country_potassium_male <- joint_sim$theta_country_potassium*matrix(1+rep(joint_sim$R_gender_potassium,52),ncol=52)/matrix(1+rep(joint_sim$R_gender_potassium,52)/2,ncol=52)

joint_sim$theta_region_sodium_female <- joint_sim$theta_region_sodium/matrix(1+rep(joint_sim$R_gender_sodium,17)/2,ncol=17)

joint_sim$theta_country_sodium_female <- joint_sim$theta_country_sodium/matrix(1+rep(joint_sim$R_gender_sodium,52)/2,ncol=52)

joint_sim$theta_region_potassium_female <- joint_sim$theta_region_potassium/matrix(1+rep(joint_sim$R_gender_potassium,17)/2,ncol=17)

joint_sim$theta_country_potassium_female <- joint_sim$theta_country_potassium/matrix(1+rep(joint_sim$R_gender_potassium,52)/2,ncol=52)

colnames(joint_sim$theta_region_potassium) <- unique(dat$Region)

colnames(joint_sim$theta_country_potassium) <- unique(dat$Country)

colnames(joint_sim$theta_country_sodium) <- unique(dat$Country)

region_data_potassium <- melt(joint_sim$theta_region_potassium)

region_data_sodium <- melt(joint_sim$theta_region_sodium)

region_data_sodium$Var2 <- region_data_potassium$Var2

region_data_ratio <- region_data_potassium

region_data_ratio$value <- (region_data_sodium$value/region_data_potassium$value)*(39/23)

country_data_potassium <- melt(joint_sim$theta_country_potassium)

country_data_sodium <- melt(joint_sim$theta_country_sodium)

country_data_ratio <- country_data_potassium

country_data_ratio$value <- (country_data_sodium$value/country_data_potassium$value)*(39/23)

####### male only calcualtions

colnames(joint_sim$theta_region_potassium_male) <- unique(dat$Region)

colnames(joint_sim$theta_country_potassium_male) <- unique(dat$Country)

colnames(joint_sim$theta_country_sodium_male) <- unique(dat$Country)

region_data_potassium_male <- melt(joint_sim$theta_region_potassium_male)

region_data_sodium_male <- melt(joint_sim$theta_region_sodium_male)

region_data_sodium_male$Var2 <- region_data_potassium_male$Var2

region_data_ratio_male <- region_data_potassium_male

region_data_ratio_male$value <- (region_data_sodium_male$value/region_data_potassium_male$value)*(39/23)

country_data_potassium_male <- melt(joint_sim$theta_country_potassium_male)

country_data_sodium_male <- melt(joint_sim$theta_country_sodium_male)

country_data_ratio_male <- country_data_potassium_male

country_data_ratio_male$value <- (country_data_sodium_male$value/country_data_potassium_male$value)*(39/23)

####### female only calculations

colnames(joint_sim$theta_region_potassium_female) <- unique(dat$Region)

colnames(joint_sim$theta_country_potassium_female) <- unique(dat$Country)

colnames(joint_sim$theta_country_sodium_female) <- unique(dat$Country)

region_data_potassium_female <- melt(joint_sim$theta_region_potassium_female)

region_data_sodium_female <- melt(joint_sim$theta_region_sodium_female)

region_data_sodium_female$Var2 <- region_data_potassium_female$Var2

region_data_ratio_female <- region_data_potassium_female

region_data_ratio_female$value <- (region_data_sodium_female$value/region_data_potassium_female$value)*(39/23)

country_data_potassium_female <- melt(joint_sim$theta_country_potassium_female)

country_data_sodium_female <- melt(joint_sim$theta_country_sodium_female)

country_data_ratio_female <- country_data_potassium_female

country_data_ratio_female$value <- (country_data_sodium_female$value/country_data_potassium_female$value)*(39/23)

joint_sim$world_wide_ratio_2_moles <- apply(acast(data = region_data_ratio,formula = iterations~Var2,fun.aggregate = NULL,value.var = "value"),1,function(x){weighted.mean(x,w=region_pop)})

joint_sim$world_wide_ratio_2_female_moles <- apply(acast(data = region_data_ratio_female,formula = iterations~Var2,fun.aggregate = NULL,value.var = "value"),1,function(x){weighted.mean(x,w=region_pop)})

joint_sim$world_wide_ratio_2_male_moles <- apply(acast(data = region_data_ratio_male,formula = iterations~Var2,fun.aggregate = NULL,value.var = "value"),1,function(x){weighted.mean(x,w=region_pop)})

joint_sim$world_wide_ratio_2_mg <- joint_sim$world_wide_ratio_2_moles*(23/39)

joint_sim$world_wide_ratio_2_female_mg <- joint_sim$world_wide_ratio_2_female_moles*(23/39)

joint_sim$world_wide_ratio_2_male_mg <- joint_sim$world_wide_ratio_2_male_moles*(23/39)

#### tables for sodium and potassium

post_summary <- function(x,digits=2){

return( paste(formatC(mean(x),digits,format='f'), " (", formatC(quantile(x,.025),digits,format='f'),",", formatC(quantile(x,.975),digits,format='f'),")",sep=''))

}

### tables

post_summary(joint_sim$world_wide_mean_sodium,digits=2)

post_summary(joint_sim$world_wide_mean_potassium,digits=2)

post_summary(joint_sim$world_wide_mean_sodium_male,digits=2)

post_summary(joint_sim$world_wide_mean_potassium_male,digits=2)

post_summary(joint_sim$world_wide_mean_sodium_female,digits=2)

post_summary(joint_sim$world_wide_mean_potassium_female,digits=2)

post_summary(joint_sim$world_wide_ratio_mg,digits=2)

post_summary(joint_sim$world_wide_ratio_female_mg,digits=2)

post_summary(joint_sim$world_wide_ratio_male_mg,digits=2)

post_summary(joint_sim$world_wide_ratio_moles,digits=2)

post_summary(joint_sim$world_wide_ratio_female_moles,digits=2)

post_summary(joint_sim$world_wide_ratio_male_moles,digits=2)

post_summary(joint_sim$world_wide_ratio_2_mg,digits=2)

post_summary(joint_sim$world_wide_ratio_2_female_mg,digits=2)

post_summary(joint_sim$world_wide_ratio_2_male_mg,digits=2)

post_summary(joint_sim$world_wide_ratio_2_moles,digits=2)

post_summary(joint_sim$world_wide_ratio_2_female_moles,digits=2)

post_summary(joint_sim$world_wide_ratio_2_male_moles,digits=2)

write.csv(country_data_ratio %>% group_by(Var2) %>%summarise(s=post_summary(value,digits=2)) %>% rename(Country=Var2, 'posterior summary'=s) ,file="../tables/no_regression/ratio_country_noregression.csv")

write.csv(region_data_ratio %>% group_by(Var2) %>%summarise(s=post_summary(value,digits=2)) %>% rename(Region=Var2, 'posterior summary'=s) ,file="../tables/no_regression/ratio_region_noregression.csv")

write.csv(country_data_sodium %>% group_by(Var2) %>%summarise(s=post_summary(value,digits=0)) %>% rename(Country=Var2, 'posterior summary'=s) ,file="../tables/no_regression/sodium_country_noregression.csv")

write.csv(region_data_sodium %>% group_by(Var2) %>%summarise(s=post_summary(value,digits=0)) %>% rename(Region=Var2, 'posterior summary'=s) ,file="../tables/no_regression/sodium_region_noregression.csv")

write.csv(country_data_potassium %>% group_by(Var2) %>%summarise(s=post_summary(value,digits=0)) %>% rename(Country=Var2, 'posterior summary'=s) ,file="../tables/no_regression/potassium_country_noregression.csv")

write.csv(region_data_potassium %>% group_by(Var2) %>%summarise(s=post_summary(value,digits=0)) %>% rename(Region=Var2, 'posterior summary'=s) ,file="../tables/no_regression/potassium_region_noregression.csv")

##### males only:

write.csv(country_data_ratio_male %>% group_by(Var2) %>%summarise(s=post_summary(value,digits=2)) %>% rename(Country=Var2, 'posterior summary'=s) ,file="../tables/no_regression/ratio_male_country_noregression.csv")

write.csv(region_data_ratio_male %>% group_by(Var2) %>%summarise(s=post_summary(value,digits=2)) %>% rename(Region=Var2, 'posterior summary'=s) ,file="../tables/no_regression/ratio_male_region_noregression.csv")

write.csv(country_data_sodium_male %>% group_by(Var2) %>%summarise(s=post_summary(value,digits=0)) %>% rename(Country=Var2, 'posterior summary'=s) ,file="../tables/no_regression/sodium_male_country_noregression.csv")

write.csv(region_data_sodium_male %>% group_by(Var2) %>%summarise(s=post_summary(value,digits=0)) %>% rename(Region=Var2, 'posterior summary'=s) ,file="../tables/no_regression/sodium_male_region_noregression.csv")

write.csv(country_data_potassium_male %>% group_by(Var2) %>%summarise(s=post_summary(value,digits=0)) %>% rename(Country=Var2, 'posterior summary'=s) ,file="../tables/no_regression/potassium_male_country_noregression.csv")

write.csv(region_data_potassium_male %>% group_by(Var2) %>%summarise(s=post_summary(value,digits=0)) %>% rename(Region=Var2, 'posterior summary'=s) ,file="../tables/no_regression/potassium_male_region_noregression.csv")

##### females only:

write.csv(country_data_ratio_female %>% group_by(Var2) %>%summarise(s=post_summary(value,digits=2)) %>% rename(Country=Var2, 'posterior summary'=s) ,file="../tables/no_regression/ratio_female_country_noregression.csv")

write.csv(region_data_ratio_female %>% group_by(Var2) %>%summarise(s=post_summary(value,digits=2)) %>% rename(Region=Var2, 'posterior summary'=s) ,file="../tables/no_regression/ratio_female_region_noregression.csv")

write.csv(country_data_sodium_female %>% group_by(Var2) %>%summarise(s=post_summary(value,digits=0)) %>% rename(Country=Var2, 'posterior summary'=s) ,file="../tables/no_regression/sodium_female_country_noregression.csv")

write.csv(region_data_sodium_female %>% group_by(Var2) %>%summarise(s=post_summary(value,digits=0)) %>% rename(Region=Var2, 'posterior summary'=s) ,file="../tables/no_regression/sodium_female_region_noregression.csv")

write.csv(country_data_potassium_female %>% group_by(Var2) %>%summarise(s=post_summary(value,digits=0)) %>% rename(Country=Var2, 'posterior summary'=s) ,file="../tables/no_regression/potassium_female_country_noregression.csv")

write.csv(region_data_potassium_female %>% group_by(Var2) %>%summarise(s=post_summary(value,digits=0)) %>% rename(Region=Var2, 'posterior summary'=s) ,file="../tables/no_regression/potassium_female_region_noregression.csv")

Accompanying Rdata file available on reasonable request to corresponding author.
