# Supplementary material for: Global mean potassium intake: a systematic review and Bayesian meta-analysis
Source: Eur J Nutr. 2023 Mar 8;62(5):2027–37. doi: 10.1007/s00394-023-03128-6 (PMC10349712; doi:10.1007/s00394-023-03128-6)
Supplement: Supplementary file 3 — Supplementary file3 (DOCX 400 KB) [file 394_2023_3128_MOESM3_ESM.docx]

# Supplementary Appendix

# **eMethods 1 – Search Strategy**

Studies were searched through September 12th, 2021.

Search terms were as follows:

(World regions) *OR* ((Afghanistan) OR (Albania) OR (Algeria) OR (Andorra) OR (Angola) OR (Antigua and Barbuda) OR (Argentina) OR (Armenia) OR (Australia) OR (Austria) OR (Azerbaijan) OR (Bahamas) OR (Bahrain) OR (Bangladesh) OR (Barbados) OR (Belarus) OR (Belgium) OR (Belize) OR (Benin) OR (Bhutan) OR (Bolivia) OR (Bosnia and Herzegovina)OR (Botswana) OR (Brazil) OR (Brunei) OR (Bulgaria) OR (Burkina Faso) OR (Burundi) OR (Cabo Verde) OR (Cambodia) OR (Cameroon) OR (Canada) OR (Central African Republic) OR (Chad) OR (Chile)OR (China) OR (Colombia) OR (Comoros) OR (Democratic Republic of the Congo) OR (Costa Rica) OR (Cote d'Ivoire)OR (Croatia) OR (Cuba) OR (Cyprus)OR (Czechia) OR (Denmark) OR (Djibouti) OR (Dominica) OR (Dominican Republic) OR (Ecuador) OR (Egypt) OR (El Salvador) OR (Equatorial Guinea) OR (Eritrea) OR (Estonia) OR (Eswatini) OR (Ethiopia) OR (Fiji) OR (Finland) OR (France) OR (Gabon) OR (Gambia) OR (Georgia) OR (Germany) OR (Ghana) OR (Greece) OR (Grenada) OR (Guatemala) OR (Guinea) OR (Guinea-Bissau) OR (Guyana) OR (Haiti) OR (Honduras) OR (Hungary) OR (Iceland) OR (India) OR (Indonesia) OR (Iran) OR (Iraq) OR (Ireland) OR (Israel) OR (Italy) OR (Jamaica) OR (Japan) OR (Jordan) OR (Kazakhstan) OR (Kenya) OR (Kiribat) OR (Kosovo) OR (Kuwait) OR (Kyrgyzstan) OR (Laos) OR (Latvia) OR (Lebanon) OR (Lesotho) OR (Liberia) OR (Libya) OR (Liechtenstein) OR (Lithuania) OR (Luxembourg) OR (Madagascar) OR (Malawi) OR (Malaysia) OR (Maldives) OR (Mali) OR (Malta) OR (Marshall Islands) OR (Mauritania) OR (Mauritius) OR (Mexico) OR (Micronesia) OR (Moldova) OR (Monaco) OR (Mongolia) OR (Montenegro) OR (Morocco) OR (Mozambique) OR (Myanmar) OR (Namibia) OR (Nauru) OR (Nepal) OR (Netherlands) OR (New Zealand) OR (Nicaragua) OR (Niger) OR (Nigeria) OR (North Korea) OR (North Macedonia) OR (Norway) OR (Oman) OR (Pakistan) OR (Palau) OR (Palestine) OR (Panama) OR (Papua New Guinea) OR (Paraguay) OR (Peru) OR (Philippines) OR (Poland) OR (Portugal) OR (Qatar) OR (Oman) OR (Romania) OR (Russia) OR (Rwanda) OR (Saint Kitts and Nevis) OR (Saint Lucia) OR (Saint Vincent and the Grenadines) OR (Samoa) OR (San Marino) OR (Sao Tome and Principe) OR (Saudi Arabia) OR (Senegal) OR (Serbia) OR (Seychelles) OR (Sierra Leone) OR (Singapore) OR (Slovakia) OR (Slovenia) OR (Solomon Islands) OR (Somalia) OR (South Africa) OR (South Korea) OR (South Sudan) OR (Spain) OR (Sri Lanka) OR (Sudan) OR (Suriname) OR (Sweden) OR (Switzerland) OR (Syria) OR (Taiwan) OR (Tajikistan) OR (Tanzania) OR (Thailand) OR (Timor-Leste) OR (Togo) OR (Tonga) OR (Trinidad and Tobago) OR (Tunisia) OR (Turkey) OR (Turkmenistan) OR (Tuvalu) OR (Uganda) OR (Ukraine) OR (United Arab Emirates) OR (United Kingdom) OR (UK) OR (United States of America) OR (Uruguay) OR (Uzbekistan) OR (Vanuatu) OR (Vatican City) OR (Venezuela) OR (Vietnam) OR (Yemen) OR (Zambia) OR (Zimbabwe))

AND

(potassium)

AND

(intake) *OR* (ingest*) *OR* (consum) *OR* (diet*) *OR* (urine) *OR* (urinary) *OR* (excret*)

NOT

Animals *NOT* Humans

# **eMethods 2 – Hierarchical joint model for per-capita sodium and potassium intake**

Estimated sample means, and standard errors, of sodium and potassium were observed for N=198 observations, 126 of which were specific to males or females and 72 of which were not gender stratified. Note that there were only S = 137 studies, each study pertaining to a single country, with some studies contributing two observations if separate male and female estimates were given. We write this raw data as:


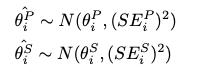


for i ∈ {1 . . . 198}, $\Theta_{i}^{P}$and $\Theta_{i}^{S}$ being the true potassium and sodium means

for the sub-populations from which $\hat{\Theta_{i}^{P}}$ and $\hat{\Theta_{i}^{S}}$ were sampled. Standard errors ${SE}_{i}^{P}$ and ${SE}_{i}^{S}$ are assumed known. While sodium estimates: $\hat{\Theta_{i}^{S}}$ were missing for 59 observations (23 studies), means $\Theta_{i}^{S}$ for these observations were naturally imputed in fitting the model. The sub-population means ($\Theta_{i}^{P}$ , $\Theta_{i}^{S}$ ) depend both on the proportion of males in the subpopulation, P_i_, and a binary indicator for whether potassium and sodium intakes were assessed using urinary measurements D_i_ = 0, or via dietary surveys, D_i_ = 1. Both these factors are assumed to have multiplicative effects on the measured outcome, denoted for potassium as (1 + $\beta_{G}^{P}P_{i}$) and (1 + $\beta_{D}^{P}D_{i}$)), with similar notation for sodium:
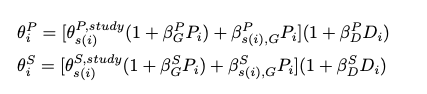
 $\Theta_{s(i)}^{P,study}$ and $\Theta_{s(i)}^{S,study}$ are the study-population means for potassium and sodium in study s(i) ∈ {1...137}, assuming measurements were made using urinary assays and on a female-only population. However, note that equation (2) allows us to convert between expected study-level potassium and sodium levels if sodium and potassium were measured using dietary surveys as op- posed to urinary assays, and for various mixtures of males and females. In the results in the main paper, we use this equation to calibrate our results to assume sodium was measured with urinary assays and potassium with dietary surveys with the estimated means corresponding to a 50% vs 50% mixture of males and females. $\beta_{s(i),G}^{S}$and $\beta_{s(i),G}^{P}$are study-level adjustments to the over-all multiplicative gender effect: (1 + $\beta_{G}^{P}P_{i}$)), with the respective adjustments for sodium and potassium having prior distributions: $\beta_{s(i),G}^{S}$∼ N(0,$\sigma_{G}^{2}$) and $\beta_{s(i),G}^{P}\sim N(0,\sigma_{G}^{2}).$ Potassium means are assumed to be linked using the following 3 level hierarchy, according to study within country, country within region and region, with independent sampling at each hierarchical level, conditional on hyper-parameters. Here, c(s) ∈ {1, ..., 52} represents the country indicator for study s, and r(c) ∈ {1, ..., 15} the region indicator for country c.


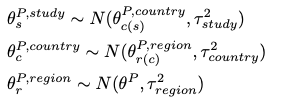


Within a country, it is assumed that study level sodium and potassium mean values are linked via a linear model:


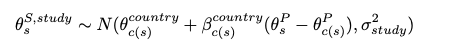


with the country-level $\Theta_{c}^{S,country}$ and region level $\Theta_{r}^{S,region}$ means for sodium

intake, and also country-level and region level sodium/potassium slopes $\beta_{c}^{country}$

and $\beta_{r}^{region}$being subject to a similar hierarchical structure:


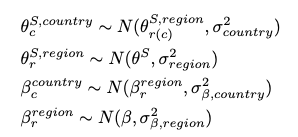


with associated variance parameters at each level.

Global potassium and sodium means were estimated by a weighted average of the estimated region level means: ${(\Theta}_{r}^{P,region},\Theta_{r}^{S,region})$ using population totals within each region as weights.

Model fitting was per- formed via Hamiltonian Monte Carlo using RStan using 10,000 iterations (the first 5,000 as warm-up) of 8 parallel chains. The following semi-informative prior distributions were set for hyper-parameters:


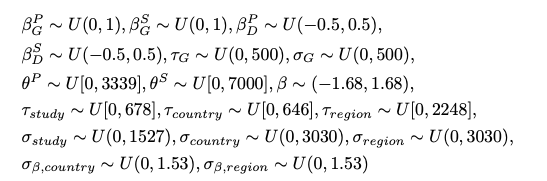


R-hat mixing values were meausred below 1.01 and the number of effective independent MCMC samples above 3,000 for all listed parameters.

## **eFigure I – Prisma flow diagram/Search Strategy**

Additional records identified through other sources
(n = 6)

Records identified through database searching
(n = 7236)

Identification

Studies included in qualitative synthesis
(n surveys = 6)

Survey/multinational cohort studies included

(n = 104 )

Records screened
(n = 6579)

Full-text articles assessed for eligibility
(n =1424)

Studies included in qualitative synthesis
(publications, n = 193, referring to surveys/multinational cohort studies, n = 98)

Full-text articles excluded, with reasons:

Potassium not reported- n=835

Sample <1000- n=432

Not representative- n=121

Conference proceeding- n=36

Records excluded
(n = 5155)

Included

Eligibility

Records after duplicates removed
(n =6579 )

Screening

**eFigure IIa – Sodium/Potassium ratio (mg derived) by Country**


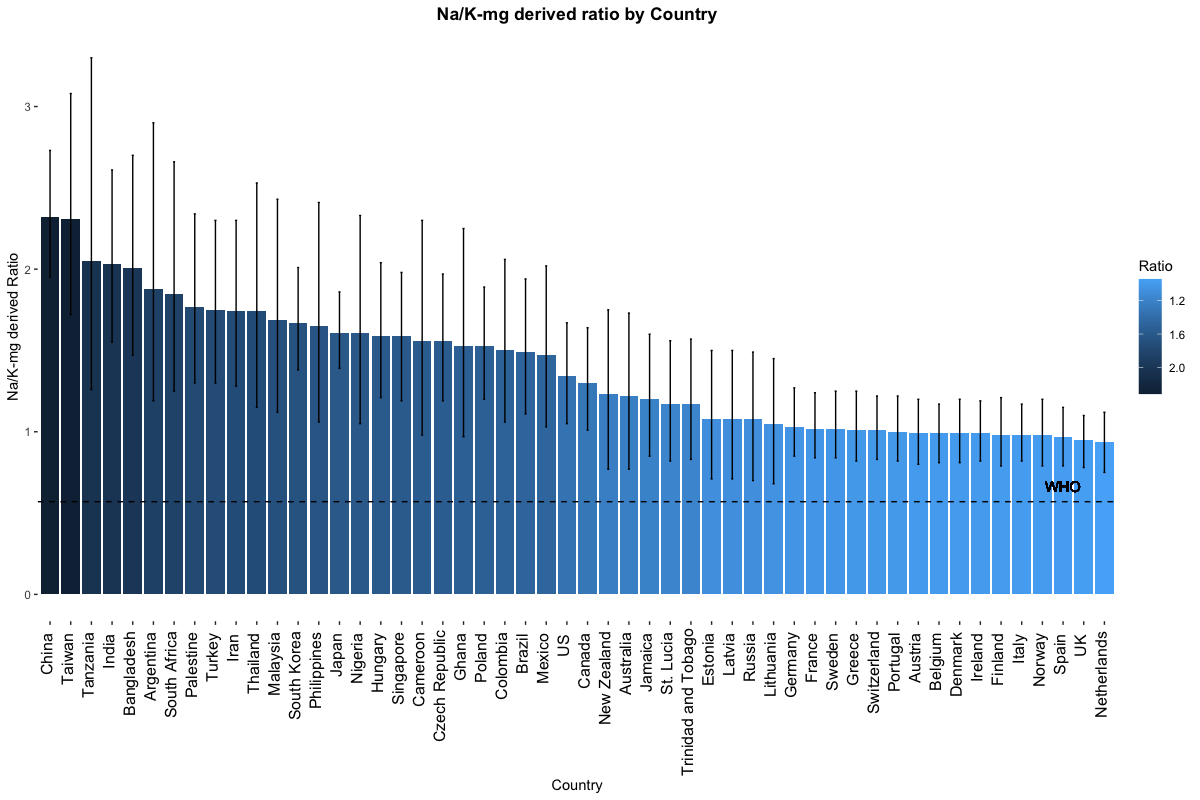


Figure IIa – Bar chart depicting mean potassium intake by country. The light blue represents higher Na/K ratio (mg/derived), with dark blue/navy representing higher Na/K ratio (mg/derived). The y-axis represents mean Na/K ratio (mg derived), ordered by Na/K ratio from highest to lowest. Error bars represent 95% credible intervals. The x-axis depicts countries included. The dashed black line represents the ratio as per WHO guidelines (0.57) for sodium and potassium in milligram units.

**eFigure IIb – Sodium/Potassium ratio estimates (mg/derived) by Country and Region**


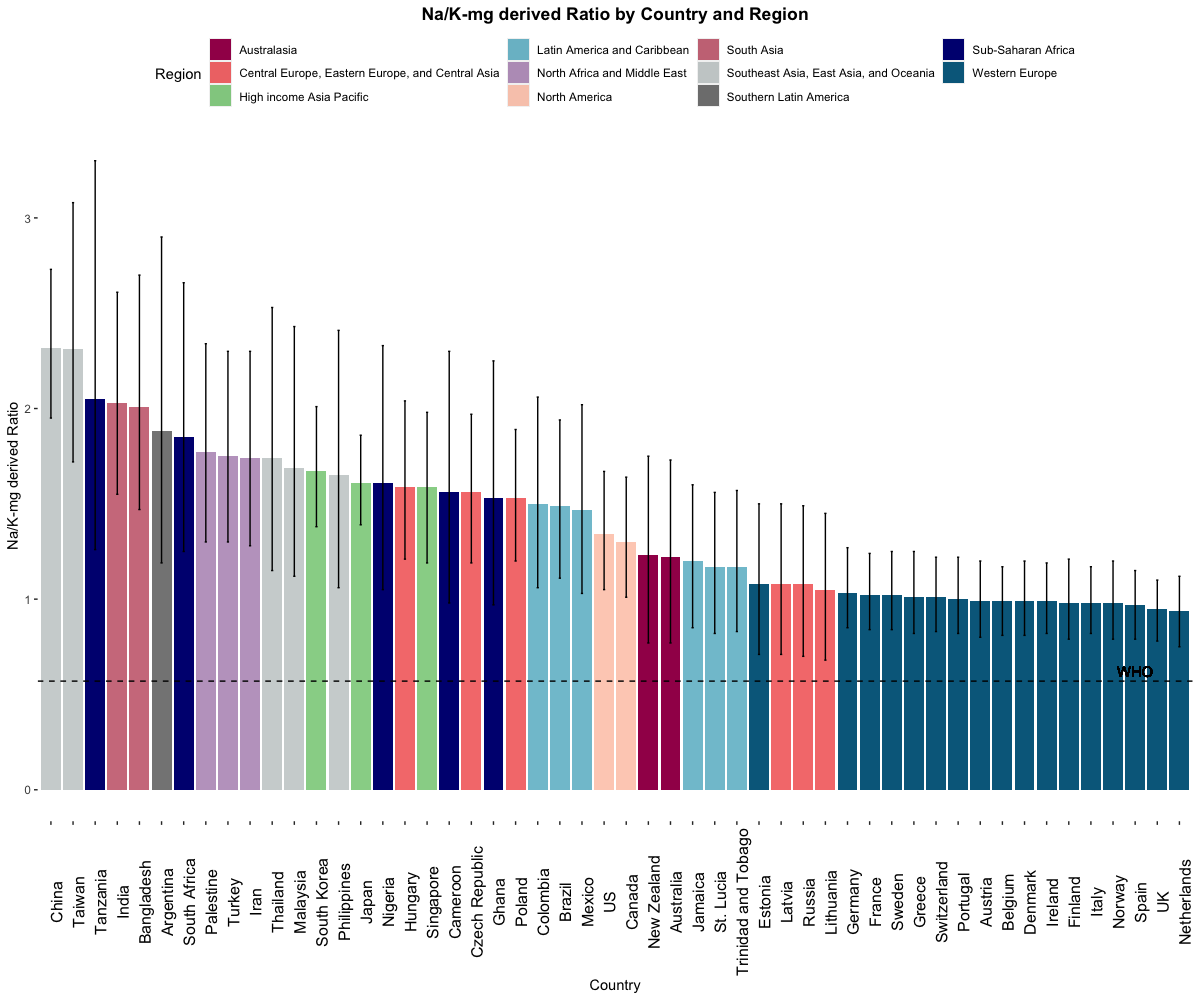


eFigure IIb – Bar chart depicting Na/K ratio by country and region. The bar colour represents regions as described in the legend; light grey- South East Asia, East Asia and Oceania, navy- Sub-Saharan Africa, pink- South Asia, dark grey- Southern Latin America, green- High income Asia Pacific, purple- North Africa and Middle East, blue- Latin America and Caribbean, peach- North America, dark red- Australasia, coral- Central/Eastern Europe and Central Asia, turquoise- Western Europe. The y-axis represents Na/K ratio. The x-axis depicts countries included. The dashed black line represents the ratio as per WHO guidelines (0.57) for sodium and potassium in milligram units.

**eFigure IIIa – Sodium/Potassium ratio (mg derived) by Country**


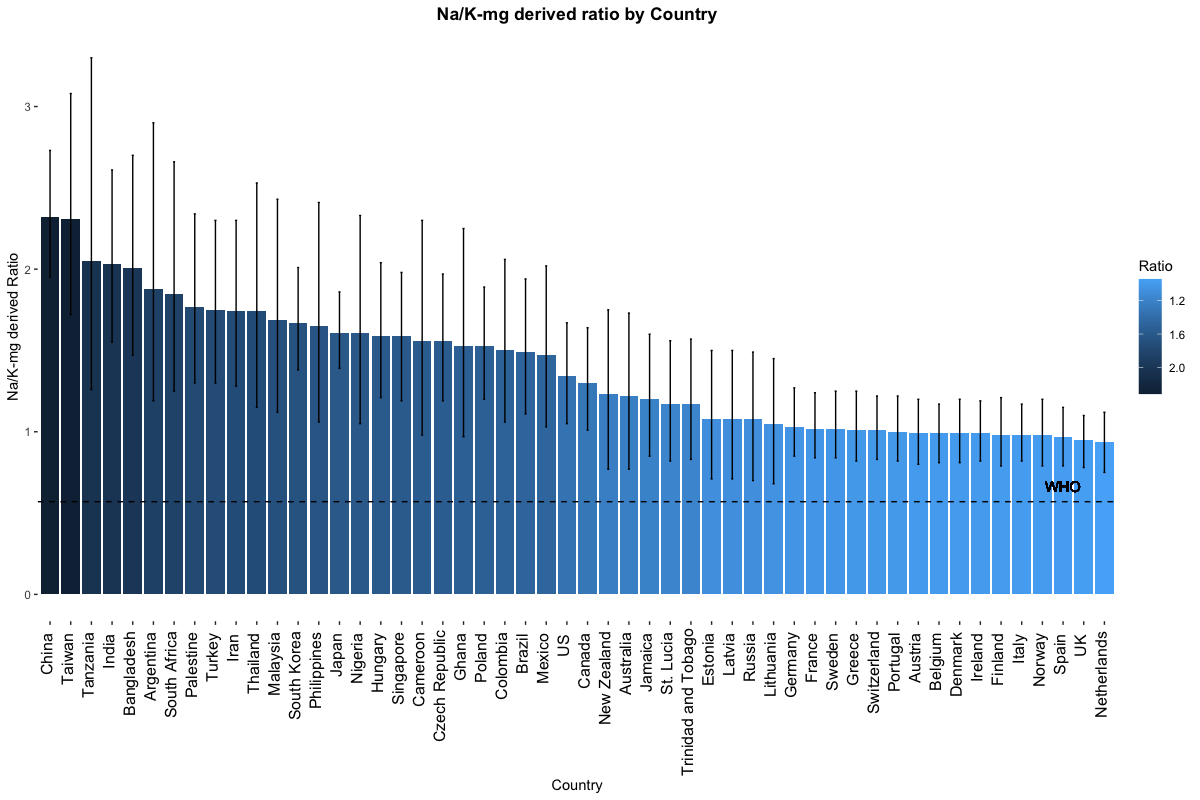


eFigure IIIa – Bar chart depicting mean potassium intake by country. The light blue represents higher Na/K ratio (mg/derived), with dark blue/navy representing higher Na/K ratio (mg/derived). The y-axis represents mean Na/K ratio (mg derived), ordered by Na/K ratio from highest to lowest. Error bars represent 95% credible intervals. The x-axis depicts countries included. The dashed black line represents the ratio as per WHO guidelines (0.57) for sodium and potassium in milligram units.

**eFigure IIIb – Sodium/Potassium ratio estimates (mg/derived) by Country and Region**


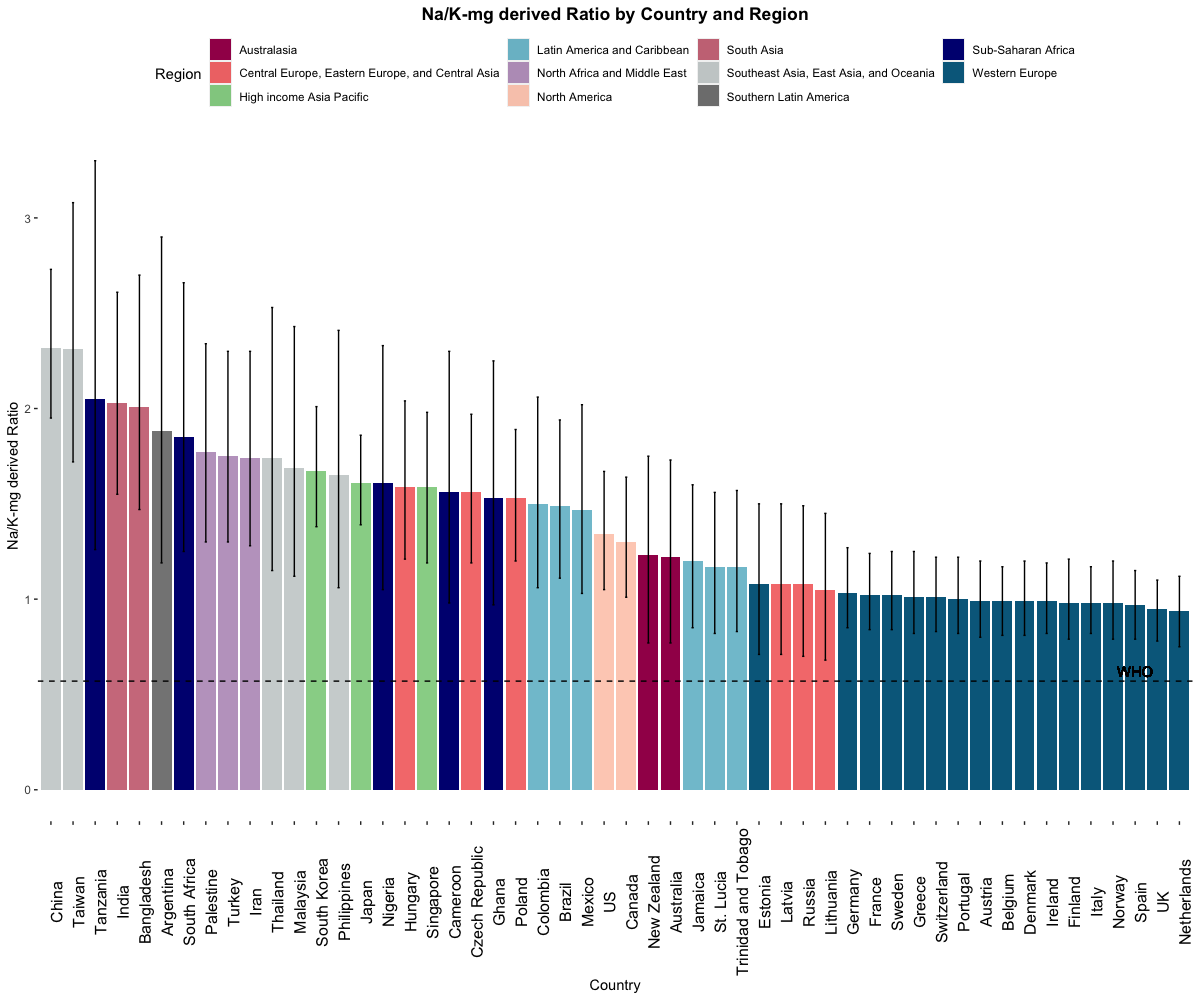


eFigure IIIb – Bar chart depicting Na/K ratio by country and region. The bar colour represents regions as described in the legend; light grey- South East Asia, East Asia and Oceania, navy- Sub-Saharan Africa, pink- South Asia, dark grey- Southern Latin America, green- High income Asia Pacific, purple- North Africa and Middle East, blue- Latin America and Caribbean, peach- North America, dark red- Australasia, coral- Central/Eastern Europe and Central Asia, turquoise- Western Europe. The y-axis represents Na/K ratio. The x-axis depicts countries included. The dashed black line represents the ratio as per WHO guidelines (0.57) for sodium and potassium in milligram units.

**eTable I – Global Burden of Disease regions, super regions**

| **Super region** | **Region** | **Countries** |
| --- | --- | --- |
| **Central Europe, Eastern Europe, and Central Asia** | | |
|  | Central Asia* | Armenia, Azerbaijan, Georgia, Kazakhstan, Kyrgyzstan, Mongolia, Tajikistan, Turkmenistan, Uzbekistan |
|  | Central Europe | Albania, Bosnia and Herzegovina, Croatia, Czech Republic , Hungary, Macedonia , Montenegro, Poland, Romania, Serbia, Slovakia, Slovenia |
|  | Eastern Europe | Belarus, Estonia, Latvia, Lithuania, Moldova , Russia, Ukraine |
| **High-income** | | |
|  | Australasia | Australia, New Zealand |
|  | High-income Asia Pacific | Brunei, Japan, Singapore, South Korea |
|  | High-income North America | Canada, United States |
|  | Southern Latin America | Argentina, Chile, Uruguay |
|  | Western Europe | Andorra, Austria, Belgium, Cyprus, Denmark, Finland, France, Germany, Greece, Greenland, Iceland, Ireland, Israel, Italy, Luxembourg, Malta, Netherlands, Norway, Portugal, Spain, Sweden, Switzerland, United Kingdom |
| **Latin America and Caribbean** | | |
|  | Andean Latin America* | Bolivia, Ecuador, Peru |
|  | Caribbean | Antigua and Barbuda, The Bahamas, Barbados, Belize, Bermuda, Cuba, Dominica, Dominican Republic, Grenada, Guyana, Haiti, Jamaica, Puerto Rico, Saint Lucia, Saint Vincent and the Grenadines, Suriname, Trinidad and Tobago |
|  | Central Latin America | Columbia, Costa Rica El Salvador, Guatemala, Honduras, Mexico, Nicaragua, Panama, Venezuela |
|  | Tropical Latin America | Brazil, Paraguay |
| **North Africa and Middle East** | | |
|  | North Africa and Middle East | Afghanistan, Algeria, Bahrain, Egypt, Iran, Iraq, Jordan, Kuwait, Lebanon, Libya, Morocco, Palestine, Oman, Qatar, Saudi Arabia, Sudan, Syria, Tunisia, Turkey, United Arab Emirates, Yemen |
| **South Asia** | | |
|  | South Asia | Bangladesh, Bhutan, India, Nepal, Pakistan |
| **Sub-Saharan Africa** | | |
|  | Central Sub-Saharan Africa* | Angola, Central African Republic, Congo, Democratic Republic of the Congo, Equatorial Guinea, Gabon |
|  | Eastern Sub-Saharan Africa | Burundi, Comoros, Djibouti, Eritrea, Ethiopia, Kenya, Madagascar, Malawi, Mozambique, Rwanda, Somalia, South Sudan, Tanzania, Uganda, Zambia |
|  | Southern Sub-Saharan Africa | Botswana, Lesotho, Namibia, South Africa, Swaziland, Zimbabwe |
|  | Western Sub-Saharan Africa | Benin, Burkina Faso, Cameroon, Cape Verde, Chad, Cote d'Ivoire, The Gambia, Ghana, Guinea, Guinea‐Bissau, Liberia, Mali, Mauritania, Niger, Nigeria, Sao Tome and Principe Senegal, Sierra Leone, Togo |
| **Southeast Asia, East Asia, and Oceania** | | |
|  | East Asia | China, North Korea, Taiwan |
|  | Southeast Asia | Cambodia, Indonesia, Laos, Malaysia, Maldives, Mauritius, Myanmar, Philippines, Seychelles, Sri Lanka, Thailand, Timor‐Leste, Vietnam |
|  | Oceania* | American Samoa, Federated States of Micronesia, Fiji, Guam, Kiribati, Marshall Islands, Papua New Guinea, Samoa, Solomon Islands, Tonga, Vanuatu |
| *No studies included for these regions | | |

## **eTable II-Classification of surveys of potassium intake by exposure metric reported, and period of survey**

|  | *Studies (n=104)* |  |  |  |  |
| --- | --- | --- | --- | --- | --- |
| **GBD region** | **Total** | **Dietary based** | **Urine based** | **Prior to 2000** | **2000-present** |
| Asia, Central | 0 | 0 | 0 | NA | NA |
| Asia, East | 16 | 5 | 11 | 5 | 11 |
| Asia Pacific high income† | 23 | 16 | 7 | 10 | 13 |
| Asia, South | 3* | 2 | 2 | 0 | 3 |
| Asia, Southeast | 3 | 2 | 1 | 0 | 3 |
| Australasia | 2 | 2 | 0 | 0 | 2 |
| Caribbean | 2 | 1 | 1 | 1 | 1 |
| Europe, Central | 7 | 6 | 1 | 0 | 5 |
| Europe, Eastern | 2 | 2 | 0 | 1 | 1 |
| Europe, Western | 37* | 29 | 10 | 17 | 20 |
| Latin America, Andean | 0 | 0 | 0 | NA | NA |
| Latin America, Central | 2 | 1 | 1 | 0 | 2 |
| Latin America, Southern | 1 | 0 | 1 | 0 | 1 |
| Latin America, Tropical | 3 | 1 | 2 | 0 | 3 |
| North America, High Income | 7 | 3 | 4 | 3 | 4 |
| North Africa Middle East | 2 | 1 | 1 | 1 | 1 |
| Oceania | 0 | 0 | 0 | NA | NA |
| Sub-Saharan Africa, East | 1 | 0 | 1 | 0 | 1 |
| Sub-Saharan Africa, Central | 0 | 0 | 0 | NA | NA |
| Sub-Saharan Africa, Southern | 2 | 0 | 2 | 0 | 2 |
| Sub-Saharan Africa, West | 3 | 0 | 3 | 1 | 2 |
| *Paired study/studies |  |  |  |  |  |

**eTable III – Potassium estimates (g/day) for Country and Region (Adults only)**

|  |  | **Overall** | **Male** | **Female** |
| --- | --- | --- | --- | --- |
| **Region** | **Country** | **Potassium intake** | **Potassium intake** | **Potassium intake** |
| **Australasia** | | 2.82 (2.06,3.60) | 3.01 (2.21,3.85) | 2.62 (1.92,3.35) |
|  | New Zealand | 2.83 (2.09,3.60) | 3.03 (2.24,3.85) | 2.63 (1.95,3.35) |
| **Caribbean** | | 2.94 (2.40,3.47) | 3.14 (2.57,3.72) | 2.73 (2.24,3.23) |
|  | Jamaica | 2.91 (2.34,3.47) | 3.11 (2.50,3.72) | 2.70 (2.18,3.23) |
|  | St. Lucia | 2.98 (2.43,3.56) | 3.19 (2.60,3.81) | 2.78 (2.26,3.31) |
|  | Trinidad and Tobago | 2.95 (2.39,3.51) | 3.15 (2.55,3.76) | 2.74 (2.22,3.26) |
| **Central Europe** | | 2.89 (2.50,3.28) | 3.10 (2.68,3.51) | 2.69 (2.33,3.05) |
|  | Czech Republic | 2.89 (2.48,3.31) | 3.10 (2.65,3.54) | 2.69 (2.30,3.08) |
|  | Hungary | 2.89 (2.43,3.33) | 3.09 (2.60,3.57) | 2.69 (2.26,3.10) |
|  | Poland | 2.92 (2.55,3.30) | 3.13 (2.73,3.53) | 2.72 (2.37,3.07) |
| **Central Latin America** | | 2.96 (2.33,3.60) | 3.16 (2.49,3.86) | 2.75 (2.17,3.35) |
|  | Colombia | 2.95 (2.30,3.59) | 3.15 (2.46,3.84) | 2.74 (2.14,3.34) |
|  | Mexico | 3.00 (2.35,3.66) | 3.21 (2.52,3.92) | 2.79 (2.19,3.40) |
| **East Asia** | | 1.89 (1.54,2.25) | 2.03 (1.64,2.41) | 1.76 (1.43,2.09) |
|  | China | 1.91 (1.65,2.16) | 2.04 (1.77,2.32) | 1.77 (1.54,2.01) |
|  | Taiwan | 1.84 (1.36,2.25) | 1.97 (1.46,2.40) | 1.71 (1.27,2.09) |
| **Eastern Europe** | | 3.54 (3.07,4.01) | 3.79 (3.28,4.30) | 3.29 (2.86,3.73) |
|  | Latvia | 3.53 (3.01,4.03) | 3.77 (3.21,4.32) | 3.28 (2.80,3.75) |
|  | Lithuania | 3.65 (3.13,4.22) | 3.91 (3.35,4.52) | 3.39 (2.91,3.92) |
|  | Russia | 3.54 (3.03,4.05) | 3.79 (3.24,4.34) | 3.29 (2.82,3.76) |
| **Eastern Sub Saharan Africa** | | 2.10 (1.32,2.86) | 2.25 (1.41,3.07) | 1.95 (1.22,2.66) |
|  | Tanzania | 2.08 (1.31,2.83) | 2.22 (1.40,3.03) | 1.93 (1.22,2.63) |
| **High income Asia Pacific** | | 2.41 (2.11,2.69) | 2.58 (2.26,2.88) | 2.24 (1.96,2.50) |
|  | Japan | 2.46 (2.24,2.68) | 2.63 (2.39,2.88) | 2.29 (2.08,2.49) |
|  | Singapore | 2.36 (1.91,2.71) | 2.53 (2.04,2.90) | 2.19 (1.77,2.52) |
|  | South Korea | 2.41 (2.11,2.70) | 2.58 (2.25,2.89) | 2.24 (1.96,2.51) |
| **High income North America** | | 2.57 (2.15,3.01) | 2.75 (2.30,3.22) | 2.39 (2.00,2.80) |
|  | Canada | 2.60 (2.16,3.05) | 2.78 (2.31,3.27) | 2.42 (2.01,2.84) |
|  | US | 2.55 (2.16,2.96) | 2.73 (2.30,3.17) | 2.37 (2.00,2.76) |
| **North Africa and Middle East** | | 2.48 (1.99,2.98) | 2.66 (2.13,3.19) | 2.31 (1.85,2.77) |
|  | Iran | 2.50 (1.97,3.04) | 2.67 (2.11,3.25) | 2.33 (1.84,2.82) |
|  | Palestine | 2.46 (1.93,2.99) | 2.63 (2.06,3.20) | 2.29 (1.79,2.78) |
|  | Turkey | 2.49 (2.00,2.99) | 2.66 (2.14,3.20) | 2.32 (1.86,2.78) |
| **South Asia** | | 1.99 (1.53,2.47) | 2.13 (1.63,2.64) | 1.85 (1.42,2.30) |
|  | Bangladesh | 2.01 (1.52,2.55) | 2.15 (1.62,2.73) | 1.87 (1.41,2.37) |
|  | India | 1.93 (1.52,2.36) | 2.07 (1.62,2.53) | 1.80 (1.41,2.20) |
| **Southeast Asia** | | 1.97 (1.40,2.53) | 2.11 (1.50,2.71) | 1.84 (1.30,2.36) |
|  | Malaysia | 2.00 (1.41,2.58) | 2.14 (1.50,2.76) | 1.86 (1.31,2.40) |
|  | Philippines | 1.95 (1.35,2.54) | 2.09 (1.44,2.72) | 1.82 (1.25,2.36) |
|  | Thailand | 1.94 (1.33,2.53) | 2.08 (1.42,2.71) | 1.81 (1.24,2.35) |
| **Southern Latin America** | | 2.26 (1.45,3.05) | 2.42 (1.55,3.26) | 2.10 (1.35,2.84) |
|  | Argentina | 2.25 (1.45,3.02) | 2.40 (1.55,3.24) | 2.09 (1.35,2.81) |
| **Southern Sub-Saharan Africa** | | 2.03 (1.34,2.67) | 2.17 (1.49,2.86) | 1.89 (1.29,2.49) |
|  | South Africa | 2.00 (1.40,2.61) | 2.14 (1.51,2.80) | 1.86 (1.30,2.43) |
| **Tropical Latin America** | | 2.70 (2.06,3.35) | 2.89 (2.20,3.59) | 2.51 (1.91,3.12) |
|  | Brazil | 2.71 (2.11,3.33) | 2.90 (2.25,3.57) | 2.52 (1.96,3.09) |
| **Western Europe** | | 3.30 (3.13,3.47) | 3.53 (3.34,3.73) | 3.07 (2.92,3.23) |
|  | Austria | 3.26 (2.89,3.55) | 3.49 (3.08,3.81) | 3.03 (2.68,3.30) |
|  | Belgium | 3.28 (2.97,3.56) | 3.51 (3.18,3.81) | 3.05 (2.76,3.31) |
|  | Denmark | 3.32 (3.03,3.62) | 3.55 (3.24,3.88) | 3.09 (2.82,3.36) |
|  | Estonia | 3.52 (3.01,4.03) | 3.77 (3.21,4.31) | 3.27 (2.79,3.75) |
|  | Finland | 3.34 (3.04,3.71) | 3.58 (3.25,3.97) | 3.11 (2.83,3.45) |
|  | France | 3.22 (2.91,3.45) | 3.44 (3.11,3.71) | 2.99 (2.71,3.21) |
|  | Germany | 3.23 (2.89,3.49) | 3.46 (3.08,3.75) | 3.00 (2.69,3.24) |
|  | Greece | 3.24 (2.89,3.51) | 3472 (3.09,3.77) | 3.02 (2.69,3.26) |
|  | Ireland | 3.29 (2.97,3.57) | 3.52 (3.18,3.83) | 3.06 (2.76,3.32) |
|  | Italy | 3.34 (3.10,3.60) | 3.57 (3.31,3.86) | 3.10 (2.88,3.35) |
|  | Netherlands | 3.40 (3.15,3.74) | 3.64 (3.36,4.00) | 3.16 (2.93,3.47) |
|  | Norway | 3.36 (3.08,3.69) | 3.59 (3.29,3.96) | 3.12 (2.86,3.43) |
|  | Portugal | 3.35 (3.04,3.72) | 3.58 (3.25,3.99) | 3.11 (2.83,3.46) |
|  | Spain | 3.31 (3.05,3.57) | 3.54 (3.25,3.83) | 3.08 (2.83,3.32) |
|  | Sweden | 3.29 (2.97,3.59) | 3.52 (3.18,3.84) | 3.06 (2.77,3.33) |
|  | Switzerland | 3.26 (2.91,3.55) | 3.49 (3.11,3.80) | 3.03 (2.71,3.30) |
|  | UK | 3.41 (3.18,3.68) | 3.65 (3.40,3.95) | 3.17 (2.96,3.42) |
| **Western Sub Saharan Africa** | | 1.83 (1.33,2.33) | 1.96 (1.42,2.49) | 1.70 (1.24,2.16) |
|  | Cameroon | 1.80 (1.26,2.34) | 1.93 (1.35,2.50) | 1.68 (1.17,2.17) |
|  | Ghana | 1.87 (1.34,2.42) | 2.00 (1.43,2.59) | 1.74 (1.25,2.25) |
|  | Nigeria | 1.78 (1.23,2.28) | 1.90 (1.35,2.45) | 1.65 (1.17,2.12) |
| Mean (95% credible intervals) | | | | |

**eTable IV – Sodium/Potassium ratio (mmol derived) estimates for Country and Region**

|  |  | **Overall** | **Male** | **Female** |
| --- | --- | --- | --- | --- |
| **Region** | **Country** | **Na/K ratio** | **Na/K ratio** | **Na/K ratio** |
| **Australasia** | | 2.10 (1.35-2.94) | 2.10 (1.36-2.95) | 2.09 (1.34-2.93) |
|  | Australia | 2.07 (1.31-2.93) | 2.07 (1.32—2.94) | 2.06 (1.31-2.91) |
|  | New Zealand | 2.09 (1.3-2.97) | 2.09 (1.31-2.99) | 2.08 (1.3-2.96) |
| **Caribbean** | | 2.01 (1.46-2.64) | 2.02 (1.46-2.65) | 2.0 (1.45-2.63) |
|  | Jamaica | 2.03 (1.43-2.72) | 2.04 (1.44-2.73) | 2.02 (1.43-2.71) |
|  | St. Lucia | 1.98 (1.4-2.66) | 2.0 (1.41-2.68) | 1.98 (1.4-2.65) |
|  | Trinidad and Tobago | 1.99 (1.4-2.66) | 2.0 (1.41-2.68) | 1.98 (1.4-2.65) |
| **Central Europe** | | 2.62 (2.04-3.27) | 2.63 (2.05-3.29) | 2.61 (2.04-3.25) |
|  | Czech Republic | 2.64 (2.02-3.34) | 2.65 (2.02-3.36) | 2.63 (2.01-3.32) |
|  | Hungary | 2.70 (2.06-3.45) | 2.71 (2.06-3.47) | 2.69 (2.05-3.44) |
|  | Poland | 2.59 (2.03-3.2) | 2.6 (2.04-3.21) | 2.58 (2.03-3.19) |
| **Central Latin America** | | 2.52 (1.8-3.42) | 2.53 (2.8-3.44) | 2.51 (1.79-3.41) |
|  | Colombia | 2.54 (1.79-3.49) | 2.55 (1.8-3.51) | 2.53 (1.79-3.47) |
|  | Mexico | 2.49 (1.74-3.42) | 2.50 (1.75-3.44) | 2.48 (1.74-3.41) |
| **East Asia** | | 3.85 (3.04-4.81) | 3.87 (3.05-4.83) | 3.84 (3.03-4.78) |
|  | China | 3.93 (3.31-4.64) | 3.95 (3.31-4.66) | 3.91 (3.30-4.62) |
|  | Taiwan | 3.92 (2.92-5.22) | 3.94 (2.92-5.24) | 3.91 (2.91-5.20) |
| **Eastern Europe** | | 1.82 (1.23-2.48) | 1.83 (1.23-2.49) | 1.81 (1.22-2.47) |
|  | Latvia | 1.83 (1.20-2.54) | 1.84 (1.20-2.55) | 1.82 (1.19-2.53) |
|  | Lithuania | 1.78 (1.16-2.46) | 1.79 (1.16-2.47) | 2.77 (1.15-2.45) |
|  | Russia | 1.82 (1.19-2.53) | 1.83 (1.19-2.54) | 1.82 (1.19-2.52) |
| **Eastern Sub Saharan Africa** | | 3.42 (2.12-5.52) | 3.43 (2.12-5.55) | 3.41 (2.11-5.49) |
|  | Tanzania | 3.48 (2.14-5.59) | 3.49 (2.15-5.61) | 3.46 (2.13-5.57) |
| **High income Asia Pacific** | | 2.75 (2.29-3.25) | 2.76 (2.3-3.27) | 2.73 (2.29-3.23) |
|  | Japan | 2.73 (2.36-3.15) | 2.75 (2.36-3.17) | 2.72 (2.35-3.13) |
|  | Singapore | 2.69 (2.01-3.36) | 2.70 (2.02-3.38) | 2.68 (2.00-3.34) |
|  | South Korea | 2.83 (2.34-3.41) | 2.84 (2.35-3.43) | 2.82 (2.33-3.39) |
| **High income North America** | | 2.26 (1.76-2.82) | 2.27 (1.77-2.84) | 2.25 (1.75-2.81) |
|  | Canada | 2.21 (1.71-2.78) | 2.22 (1.71-2.80) | 2.20 (1.70-2.76) |
|  | US | 2.28 (1.78-2.84) | 2.29 (1.78-2.86) | 2.27 (1.77-2.83) |
| **North Africa and Middle East** | | 2.95 (2.22-3.85) | 2.96 (2.23-3.87) | 2.93 (2.21-3.83) |
|  | Iran | 2.94 (2.17-3.89) | 2.95 (2.18-3.91) | 2.93 (2.16-3.88) |
|  | Palestine | 3.00 (2.21-3.90) | 2.01 (2.21-3.99) | 2.98 (2.20-3.97) |
|  | Turkey | 2.97 (2.21-3.90) | 2.98 (2.21-3.92) | 2.95 (2.19-3.88) |
| **South Asia** | | 3.39 (2.54-4.45) | 3.4 (2.55-4.47) | 3.37 (2.54-4.44) |
|  | Bangladesh | 3.41 (2.49-4.58) | 3.42 (2.50-4.61) | 3.39 (2.48-4.57) |
|  | India | 3.44 (2.62-4.43) |  |  |
| **Southeast Asia** | | 2.87 (1.95-4.06) | 2.88 (1.95-4.08) | 2.86 (1.94-4.04) |
|  | Malaysia | 2.86 (1.90-4.12) | 2.87 (1.91-4.15) | 2.85 (1.89-4.11) |
|  | Philippines | 2.80 (1.80-4.08) | 2.81 (1.80-4.09) | 2.79 (1.79-4.06) |
|  | Thailand | 2.95 (1.95-4.29) | 2.96 (1.95-4.31) | 2.93 (1.94-4.27) |
| **Southern Latin America** | | 3.14 (2.01-4.89) | 3.15 (2.01-4.91) | 3.13 (2.0-4.88) |
|  | Argentina | 3.19 (2.02-4.91) | 3.20 (2.03-4.93) | 3.17 (2.02-4.90) |
| **Southern Sub-Saharan Africa** | | 3.12 (2.09-4.55) | 3.13 (2.1-4.56) | 3.1 (2.08-4.54) |
|  | South Africa | 3.13 (2.12-4.51) | 3.15 (2.13-4.53) | 3.12 (2.11-4.49) |
| **Tropical Latin America** | | 2.52 (1.85-3.35) | 2.53 (1.86-3.37) | 2.51 (1.84-3.34) |
|  | Brazil | 2.52 (1.88-3.29) | 2.53 (1.88-3.30) | 2.51 (1.87-3.27) |
| **Western Europe** | | 1.68 (1.46-1.92) | 1.69 (1.46-1.94) | 1.68 (1.46-1.91) |
|  | Austria | 1.68 (1.35-2.04) | 1.69 (1.35-2.06) | 1.68 (1.34-2.03) |
|  | Belgium | 1.67 (1.38-1.99) | 1.68 (1.38-2.00) | 1.66 (1.37-1.98) |
|  | Denmark | 1.68 (1.37-2.04) | 1.69 (1.37-2.05) | 1.68 (1.37-2.03) |
|  | Estonia | 1.83 (1.20-2.54) | 1.84 (1.20-2.55) | 1.82 (1.19-2.53) |
|  | Finland | 1.67 (1.34-2.05) | 1.68 (1.38-2.06) | 1.66 (1.33-2.04) |
|  | France | 1.74 (1.43-2.10) | 1.74 (1.44-2.17) | 1.73 (1.42-2.08) |
|  | Germany | 1.75 (1.44-2.15) | 1.76 (1.44-2.17) | 1.74 (1.44-2.14) |
|  | Greece | 1.72 (1.39-2.11) | 1.72 (1.39-2.12) | 1.71 (1.38-2.10) |
|  | Ireland | 1.68 (1.38-2.02) | 1.69 (1.38-2.04) | 1.68 (1.38-2.01) |
|  | Italy | 1.67 (1.38-1.98) | 1.67 (1.39-1.99) | 1.66 (1.38-1.97) |
|  | Netherlands | 1.60 (1.27-1.90) | 1.60 (1.27-1.91) | 1.59 (1.27-1.89) |
|  | Norway | 1.67 (1.34-2.04) | 1.67 (1.34-2.05) | 1.66 (1.34-2.03) |
|  | Portugal | 1.69 (1.39-2.07) | 1.70 (1.39-2.08) | 1.69 (1.38-2.06) |
|  | Spain | 1.64 (1.34-1.95) | 1.65 (1.34-1.96) | 1.64 (1.33-1.94) |
|  | Sweden | 1.72 (1.42-2.12) | 1.73 (1.42-2.13) | 1.72 (1.41-2.11) |
|  | Switzerland | 1.71 (1.41-2.07) | 1.72 (1.41-2.08) | 1.70 (1.40-2.06) |
|  | UK | 1.60 (1.33-1.87) | 1.72 (1.41-2.08) | 1.70 (1.40-2.06) |
| **Western Sub-Saharan Africa** | | 2.68 (1.76-3.82) | 2.69 (1.76-3.84) | 2.67 (1.75-3.81) |
|  | Cameroon | 2.64 (1.66-3.89) | 2.65 (1.66-3.91) | 2.63 (1.65-3.88) |
|  | Ghana | 2.60 (1.64-3.81) | 2.61 (1.64-3.83) | 2.59 (1.64-3.79) |
|  | Nigeria | 2.73 (1.77-3.95) | 2.74 (1.78-3.96) | 2.72 (1.76-3.93) |
| **Global** | | *2.88 (2.56-3.23) |  |  |
| *Calculated as weighted global mean sodium and potassium. Global ratio calculated as weighted average of mean ratios within region- 3.02 (2.65-3.45)  Mean (95% credible intervals) | | | | |

**eTable V – Sodium/Potassium ratio (mg/derived) estimates for Country and Region**

|  |  | **Overall** | **Male** | **Female** |
| --- | --- | --- | --- | --- |
| **Region** | **Country** | **Na/K ratio** | **Na/K ratio** | **Na/K ratio** |
| **Australasia** | | 1.24 (0.80,1.73) | 1.24 (0.80,1.74) | 1.23 (0.79,1.73) |
|  | Australia | 1.22 (0.77,1.73) | 1.22 (0.78,1.73) | 1.21 (0.77,1.72) |
|  | New Zealand | 1.23 (0.77,1.75) | 1.24 (0.77,1.76) | 1.23 (0.76,1.75) |
| **Caribbean** | | 1.19 (0.86,1.56) | 1.19 (0.86,1.56) | 1.18 (0.86,1.55) |
|  | Jamaica | 1.20 (0.85,1.60) | 1.20 (0.85,1.61) | 1.19 (0.84,1.60) |
|  | St. Lucia | 1.17 (0.82,1.56) | 1.17 (0.82,1.57) | 1.16 (0.82,1.56) |
|  | Trinidad and Tobago | 1.17 (0.83,1.57) | 1.18 (0.83,1.58) | 1.17 (0.82,1.56) |
| **Central Europe** | | 1.55 (1.21,1.93) | 1.55 (1.21,1.94) | 1.54 (1.20,1.92) |
|  | Czech Republic | 1.56 (1.19,1.97) | 1.57 (1.19,1.98) | 1.55 (1.19,1.96) |
|  | Hungary | 1.59 (1.21,2.04) | 1.60 (1.22,2.05) | 1.58 (1.21,2.03) |
|  | Poland | 1.53 (1.20,1.89) | 1.54 (1.20,1.90) | 1.52 (1.19,1.88) |
| **Central Latin America** | | 1.48 (1.06,2.02) | 1.49 (1.06,2.03) | 1.48 (1.06,2.01) |
|  | Colombia | 1.50 (1.06,2.06) | 1.51 (1.06,2.07) | 1.49 (1.05,2.05) |
|  | Mexico | 1.47 (1.03,2.02) | 1.48 (1.03,2.03) | 1.46 (1.03,2.01) |
| **East Asia** | | 2.27 (1.79,2.83) | 2.28 (1.80,2.85) | 2.26 (1.79,2.82) |
|  | China | 2.32 (1.95,2.73) | 2.33 (1.95,2.75) | 2.31 (1.95,2.73) |
|  | Taiwan | 2.31 (1.72,3.08) | 2.32 (1.72,3.09) | 2.30 (1.71,3.06) |
| **Eastern Europe** | | 1.07 (0.73,1.46) | 1.08 (0.73,1.47) | 1.07 (0.72,1.46) |
|  | Latvia | 1.08 (0.71,1.50) | 1.08 (0.71,1.51) | 1.07 (0.70,1.49) |
|  | Lithuania | 1.05 (0.68,1.45) | 1.05 (0.68,1.46) | 1.04 (0.68,1.45) |
|  | Russia | 1.08 (0.70,1.49) | 1.08 (0.70,1.50) | 1.07 (0.70,1.48) |
| **Eastern Sub Saharan Africa** | | 2.02 (1.25,3.26) | 2.03 (1.25,3.28) | 2.01 (1.24,3.24) |
|  | Tanzania | 2.05 (1.26,3.30) | 2.06 (1.27,3.31) | 2.04 (1.26,3.28) |
| **High income Asia Pacific** | | 1.62 (1.35,1.92) | 1.62 (1.35,1.93) | 1.61 (1.35,1.91) |
|  | Japan | 1.61 (1.39,1.86) | 1.62 (1.39,1.87) | 1.61 (1.39,1.85) |
|  | Singapore | 1.59 (1.19,1.98) | 1.59 (1.19,1.99) | 1.58 (1.18,1.97) |
|  | South Korea | 1.67 (1.38,2.01) | 1.68 (1.38,2.02) | 1.66 (1.38,2.00) |
| **High income North America** | | 1.33 (1.04,1.66) | 1.34 (1.04,1.67) | 1.33 (1.03,1.66) |
|  | Canada | 1.30 (1.01,1.64) | 1.31 (1.01,1.65) | 1.30 (1.00,1.63) |
|  | US | 1.34 (1.05,1.67) | 1.35 (1.05,1.69) | 1.34 (1.04,1.67) |
| **North Africa and Middle East** | | 1.74 (1.31,2.27) | 1.74 (1.32,2.28) | 1.73 (1.30,2.26) |
|  | Iran | 1.74 (1.28,2.30) | 1.74 (1.28,2.30) | 1.73 (1.28,2.29) |
|  | Palestine | 1.77 (1.30,2.34) | 1.77 (1.31,2.35) | 1.76 (1.30,2.34) |
|  | Turkey | 1.75 (1.30,2.30) | 1.76 (1.31,2.31) | 1.74 (1.29,2.29) |
| **South Asia** | | 2.00 (1.50,2.62) | 2.00 (1.50,2.64) | 1.99 (1.50,2.62) |
|  | Bangladesh | 2.01 (1.47,2.70) | 2.02 (1.47,2.72) | 2.00 (1.46,2.69) |
|  | India | 2.03 (1.55,2.61) | 2.04 (1.55,2.63) | 2.02 (1.54,2.60) |
| **Southeast Asia** | | 1.69 (1.15,2.39) | 1.70 (1.15,2.41) | 1.69 (1.14,2.38) |
|  | Malaysia | 1.69 (1.12,2.43) | 1.69 (1.13,2.45) | 1.68 (1.12,2.42) |
|  | Philippines | 1.65 (1.06,2.41) | 1.66 (1.06,2.41) | 1.65 (1.06,2.39) |
|  | Thailand | 1.74 (1.15,2.53) | 1.74 (1.15,2.54) | 1.73 (1.14,2.52) |
| **Southern Latin America** | | 1.85 (1.18,2.88) | 1.86 (1.19,2.90) | 1.85 (1.18,2.88) |
|  | Argentina | 1.88 (1.19,2.90) | 1.89 (1.20,2.91) | 1.87 (1.19,2.89) |
| **Southern Sub Saharan Africa** | | 1.84 (1.23,2.69) | 1.84 (1.24,2.69) | 1.83 (1.23,2.68) |
|  | South Africa | 1.85 (1.25,2.66) | 1.86 (1.26,2.67) | 1.84 (1.25,2.65) |
| **Tropical Latin America** | | 1.49 (1.09,1.98) | 1.49 (1.10,1.99) | 1.48 (1.09,1.97) |
|  | Brazil | 1.49 (1.11,1.94) | 1.49 (1.11,1.95) | 1.48 (1.10,1.93) |
| **Western Europe** | | 0.99 (0.86,1.13) | 1.00 (0.86,1.14) | 0.99 (0.86,1.13) |
|  | Austria | 0.99 (0.80,1.20) | 1.00 (0.80,1.21) | 0.99 (0.79,1.20) |
|  | Belgium | 0.99 (0.81,1.17) | 0.99 (0.81,1.18) | 0.98 (0.81,1.17) |
|  | Denmark | 0.99 (0.81,1.20) | 1.00 (0.81,1.21) | 0.99 (0.81,1.20) |
|  | Estonia | 1.08 (0.71,1.50) | 1.08 (0.71,1.50) | 1.08 (0.70,1.49) |
|  | Finland | 0.98 (0.79,1.21) | 0.99 (0.79,1.22) | 0.98 (0.79,1.20) |
|  | France | 1.02 (0.84,1.24) | 1.03 (0.84,1.24) | 1.02 (0.84,1.23) |
|  | Germany | 1.03 (0.85,1.27) | 1.04 (0.85,1.28) | 1.03 (0.85,1.26) |
|  | Greece | 1.01 (0.82,1.25) | 1.02 (0.82,1.25) | 1.01 (0.82,1.24) |
|  | Ireland | 0.99 (0.82,1.19) | 1.00 (0.82,1.20) | 0.99 (0.81,1.19) |
|  | Italy | 0.98 (0.82,1.17) | 0.99 (0.82,1.18) | 0.98 (0.81,1.16) |
|  | Netherlands | 0.94 (0.75,1.12) | 0.95 (0.75,1.13) | 0.94 (0.75,1.12) |
|  | Norway | 0.98 (0.79,1.20) | 0.99 (0.79,1.21) | 0.98 (0.79,1.20) |
|  | Portugal | 1.00 (0.82,1.22) | 1.00 (0.82,1.23) | 0.99 (0.82,1.22) |
|  | Spain | 0.97 (0.79,1.15) | 0.97 (0.79,1.16) | 0.97 (0.78,1.14) |
|  | Sweden | 1.02 (0.84,1.25) | 1.02 (0.84,1.26) | 1.01 (0.83,1.24) |
|  | Switzerland | 1.01 (0.83,1.22) | 1.01 (0.83,1.23) | 1.00 (0.83,1.22) |
|  | UK | 0.95 (0.78,1.10) | 0.95 (0.78,1.11) | 0.94 (0.78,1.10) |
| **Western Sub Saharan Africa** | | 1.58 (1.04,2.26) | 1.58 (1.04,2.26) | 1.57 (1.03,2.25) |
|  | Cameroon | 1.56 (0.98,2.30) | 1.56 (0.98,2.30) | 1.55 (0.97,2.29) |
|  | Ghana | 1.53 (0.97,2.25) | 1.54 (0.97,2.26) | 1.53 (0.96,2.24) |
|  | Nigeria | 1.61 (1.05,2.33) | 1.62 (1.05,2.34) | 1.60 (1.04,2.32) |
| **Global** | | *1.70 (1.51-1.90) |  |  |
| *Calculated as weighted global mean sodium and potassium. Global ratio calculated as weighted average of mean ratios within region- 1.78 (1.56-2.03)  Mean (credible interval) | | | | |

**eTable VI – GATHER checklist**

| Item # | Checklist item | Reported on page # |
| --- | --- | --- |
| Objectives and funding | | |
| 1 | Define the indicator(s), populations (including age, sex, and geographic entities), and time period(s) for which estimates were made. | Abstract, 8 |
| 2 | List the funding sources for the work. | 19 |
| Data Inputs | | |
| *For all data inputs from multiple sources that are synthesized as part of the study:* | | |
| 3 | Describe how the data were identified and how the data were accessed. | 9 |
| 4 | Specify the inclusion and exclusion criteria. Identify all ad-hoc exclusions. | 9 |
| 5 | Provide information on all included data sources and their main characteristics. For each data source used, report reference information or contact name/institution, population represented, data collection method, year(s) of data collection, sex and age range, diagnostic criteria or measurement method, and sample size, as relevant. | 11, Table 2 (20-21) |
| 6 | Identify and describe any categories of input data that have potentially important biases (e.g., based on characteristics listed in item 5). | 20-21 |
| *For data inputs that contribute to the analysis but were not synthesized as part of the study:* | | |
| 7 | Describe and give sources for any other data inputs. | NA |
| *For all data inputs:* | | |
| 8 | Provide all data inputs in a file format from which data can be efficiently extracted (e.g., a spreadsheet rather than a PDF), including all relevant meta-data listed in item 5. For any data inputs that cannot be shared because of ethical or legal reasons, such as third-party ownership, provide a contact name or the name of the institution that retains the right to the data. | Supplementary appendix 2 |
| Data analysis | | |
| 9 | Provide a conceptual overview of the data analysis method. A diagram may be helpful. | 10-11, eMethods |
| 10 | Provide a detailed description of all steps of the analysis, including mathematical formulae. This description should cover, as relevant, data cleaning, data pre-processing, data adjustments and weighting of data sources, and mathematical or statistical model(s). | eMethods |
| 11 | Describe how candidate models were evaluated and how the final model(s) were selected. | eMethods |
| 12 | Provide the results of an evaluation of model performance, if done, as well as the results of any relevant sensitivity analysis. |  |
| 13 | Describe methods for calculating uncertainty of the estimates. State which sources of uncertainty were, and were not, accounted for in the uncertainty analysis. | 10-11,eMethods |
| 14 | State how analytic or statistical source code used to generate estimates can be accessed. | Supplementary appendix 3 |
| Results and Discussion | | |
| 15 | Provide published estimates in a file format from which data can be efficiently extracted. | 22-25 |
| 16 | Report a quantitative measure of the uncertainty of the estimates (e.g. uncertainty intervals). | 22-25 |
| 17 | Interpret results in light of existing evidence. If updating a previous set of estimates, describe the reasons for changes in estimates. | 14-17 |
| 18 | Discuss limitations of the estimates. Include a discussion of any modelling assumptions or data limitations that affect interpretation of the estimates. | 17-18 |
